# Altered hepatic genes related to retinol metabolism and plasma retinol in patients with non-alcoholic fatty liver disease

**DOI:** 10.1371/journal.pone.0205747

**Published:** 2018-10-31

**Authors:** Paulina Pettinelli, Bianca M. Arendt, Anastasia Teterina, Ian McGilvray, Elena M. Comelli, Scott K. Fung, Sandra E. Fischer, Johane P. Allard

**Affiliations:** 1 Department of Health Sciences, Nutrition and Dietetics, Faculty of Medicine, Pontificia Universidad Católica de Chile, Santiago, Chile; 2 Toronto General Hospital, University Health Network, Toronto, Ontario, Canada; 3 Department of Medicine, University of Toronto, Toronto, Ontario, Canada; 4 Department of Nutritional Sciences and Centre for Child Nutrition and Health, University of Toronto, Toronto, Ontario, Canada; 5 Department of Laboratory Medicine and Pathobiology, University of Toronto, Toronto, Ontario, Canada; University College London, UNITED KINGDOM

## Abstract

Non-alcoholic fatty liver disease (NAFLD), especially non-alcoholic steatohepatitis (NASH) is a chronic liver disease commonly associated with hepatic fibrosis. NASH patients have an increased risk for hepatocellular carcinoma (HCC). An altered retinol metabolism is one of the pathways involved in the process of hepatic fibrosis, and enzymes involved in retinol metabolism have been associated with HCC. We aimed to determine the association between plasma retinol levels and hepatic expression of genes related to retinol metabolism, as well as to assess the hepatic expression of transcription factors regulated by retinoic acid in patients with NAFLD. Cross-sectional study where hepatic gene expression (Illumina microarray) and plasma retinol levels (HPLC) were measured in 17 patients with simple steatosis (SS), 15 with NASH, and 22 living liver donors (LD) as controls. Plasma retinol levels were higher in SS (1.53 ± 0.44 μmol/L) and NASH (1.51 ± 0.56 μmol/L) compared to LD (1.21 ± 0.38 μmol/L; p<0.05). *AKR1B10* was highly overexpressed in NASH compared to SS (+6.2-fold) and LD (+9.9-fold; p = 4.89E-11). Retinaldehyde dehydrogenase 1 family, member A2 (*ALDH1A2*) and retinaldehyde dehydrogenase 1 family, member A3 (*ALDH1A3*), key enzymes for retinoic acid synthesis, were underexpressed in SS (-1.48 and -2.3-fold, respectively) and NASH (-1.47 and -2.6-fold, respectively) versus LD. In NASH, hepatic *ALDH1A2* and *ALDH1A3* were underexpressed and inversely correlated with plasma retinol levels, which may reduce retinoic acid in the liver. This, in addition to changes in expression of other genes involved in retinol metabolism, suggests a role for altered retinol homeostasis in NASH.

## Introduction

Patients with non-alcoholic fatty liver disease (NAFLD), especially those with non-alcoholic steatohepatitis (NASH), have an increased risk for hepatocellular carcinoma (HCC), even in the absence of cirrhosis.[1–4] In addition, NASH is one of the chronic liver diseases commonly associated with hepatic fibrosis.[5]

**Fig 1:**
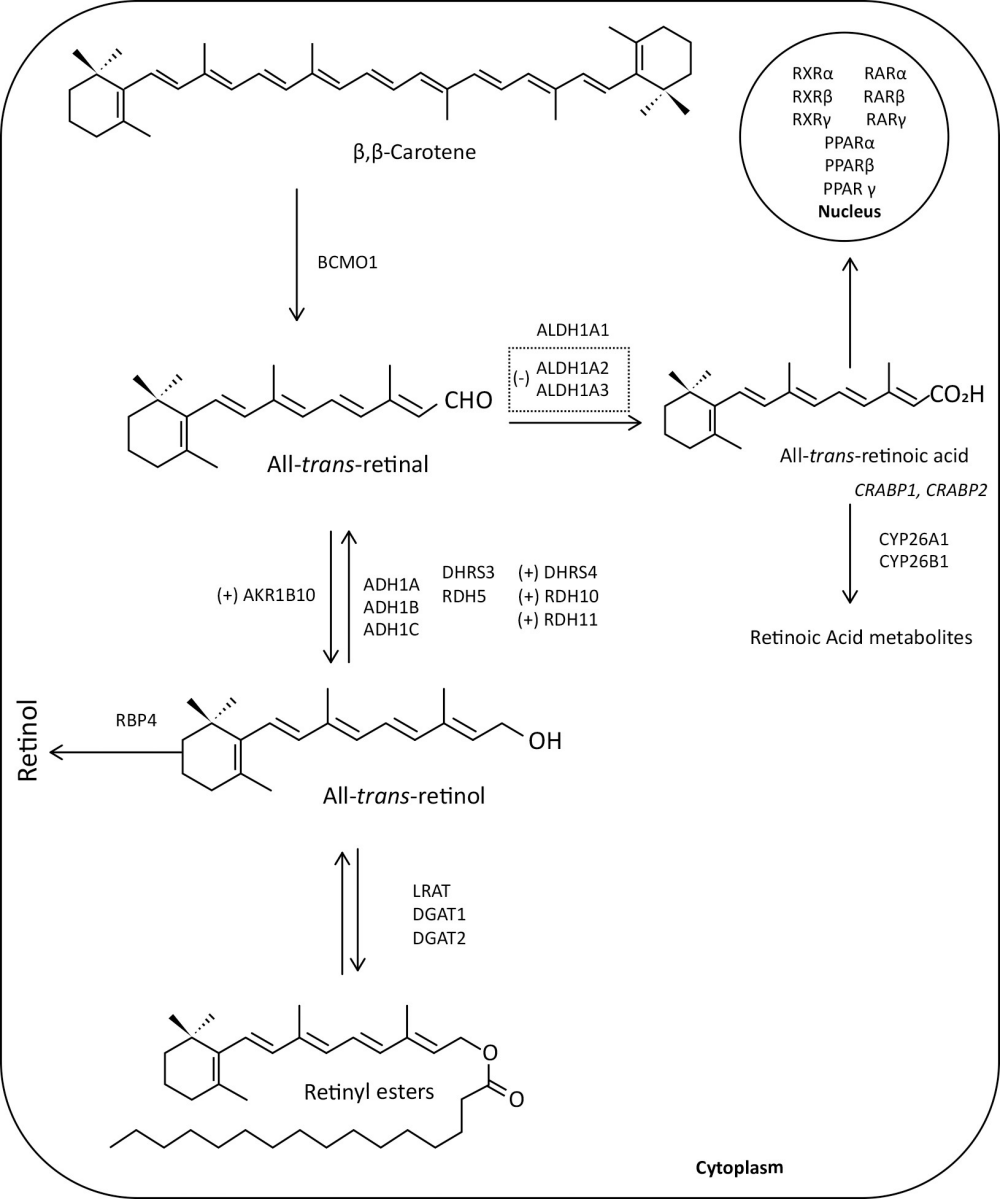
Intracellular metabolism of retinol (vitamin A) in the liver. Retinol is provided by our diet and transported via chylomicrons into the liver, where it is bound to retinol binding protein (RBP). Retinol is converted to retinyl ester by lecithin retinol acyltransferase (LRAT), for storage. Retinol is converted into all-trans-retinal in the liver via two oxidative steps. The first enzymatic step is the reversible oxidation of retinol to retinal by three types of enzymes: i) members of retinal dehydrogenase family (RDH5, RDH10, RDH11), ii) alcohol dehydrogenase (ADH1, ADH1B and ADH1C); and iii) membrane-bound short-chain dehydrogenases/reductases (DHRS3 and DHRS4), all involved in maintaining an equilibrium between retinal and retinol. The second and irreversible step is the oxidation of retinal to all-trans-retinoic acid by retinaldehyde dehydrogenase 1 family, member A1, A2 and A3 (ALDH1A1, ALDH1A2 and ALDH1A3). Excessive all-trans-retinoic acid, which is bound to cellular retinoic acid-binding proteins (CRABP1 and CRABP2), is not recycled back to retinol and must be oxidized to be eliminated from the body by the cytochrome P450 family members CYP26A1 and CYP26B1. Newly synthesized all-trans-retinoic acid can be bound to CRABP2. All-trans-retinoic acid that enters the nucleus, binds to a retinoic acid receptor/retinoic X receptor (RAR/RXR) heterodimer and stimulates transcription of target genes. The plus sign (+) indicates increased expression of the enzyme AKR1B10, whereas the minus sign (-) indicates a reduced expression of the enzymes ALDH1A2 and ALDH1A3.

The term “vitamin A” is a generic descriptor for compounds that have the biological activity of retinol or its metabolic products. Retinol is an essential nutrient for humans and must be provided by the diet.[6,7] Retinyl esters in chylomicrons enter the circulation and are taken up by tissues as the chylomicron undergoes lipolysis and remodeling. Approximately 66–75% of chylomicron retinyl ester is cleared by the liver, and the remainder is cleared by peripheral tissues.[8] Once in the blood, retinol forms a complex with retinol binding protein 4 (RBP4), and inside the cell it is metabolized and converted to retinoic acid by various enzymes via a two-step oxidation process (Fig 1).[8] All-*trans* retinoic acid is the main endogenous active retinoic acid metabolite. It regulates specific nuclear receptors (retinoic acid receptors, RAR, and retinoid X receptors, RXR) which in turn influence the expression of several genes that play a role in cell growth, differentiation, development, and homeostasis.[9,10]

In a healthy liver, hepatic stellate cells (HSC) maintain a non-proliferative, quiescent phenotype storing 70–80% of total liver retinoid (vitamin A).[8,11] When HSC become activated, they lose their intracellular vitamin A storage, which leads to the transformation into collagen producing myofibroblasts.[5,12]

An altered retinol metabolism has been identified as one of the different pathways involved in the complex process of hepatic fibrosis.[5] Furthermore, it has been reported that aldo-keto reductase family 1 member B10 (*AKR1B10*), which is a key enzyme of the retinol metabolism with a very efficient high all-*trans*-retinaldehyde reductase activity, converting all-*trans*-retinaldehyde to retinol,[13,14] is overexpressed in primary malignant liver tumors compared to non-tumorous cirrhotic liver tissue, especially in early stages, as well as in other cancer types and in pre-cancerous lesions (Fig 1).[15,16] Moreover, two cross-sectional studies demonstrated increased *AKR1B10* expression in the liver of patients with NASH compared to those with simple hepatic steatosis and controls,[17,18] suggesting that *AKR1B10* may be a potential biomarker for NASH and progression to HCC.

Reports related to vitamin A intake and retinol metabolism in NAFLD patients are scarce and show varying results. In a previous study, we reported adequate vitamin A intake in patients with NAFLD, which was similar to healthy controls,[19] while others found lower intakes in NAFLD.[20] Patients with NAFLD had also higher serum retinol levels [21] and higher plasma RBP4 levels compared to control subjects,[22,23] whereas RBP4 was not different between simple steatosis (SS) and NASH.[24–26] Another report showed that hepatic retinol reserves in patients with NASH were lower compared to those with SS.[27]

Considering that the liver is the most important organ for the storage and metabolism of retinol containing the enzymes for retinol metabolism,[11] we wanted to investigate, whether there is an association between a disturbed retinol metabolism and disease severity in NAFLD [28] and explore the potential role of genes related to retinol metabolism. The aim of this study was to a) determine the relationship between plasma retinol levels and hepatic expression of genes related to retinol metabolism in patients with NAFLD (SS or NASH) and healthy living liver donors as controls, as well as to b) assess the hepatic expression of transcription factors regulated by retinoic acid.

## Materials and methods

This was a cross-sectional study conducted at the University Health Network, Toronto, Canada; see S1 and S2 Files. Details of the study have been described previously.[18] Patients and controls were recruited between March 2007 and November 2011 from the Hepatology clinic and the Multiorgan Transplant Program respectively, at the University Health Network, Toronto, Canada. The study was approved by the University Health Network Research Ethics Board (REB# 03-0505-A), was registered (NCT02148471, www.clinicaltrials.gov), and was performed in accordance with the 1975 Declaration of Helsinki and its revisions. All participants gave their written informed consent.

### Study participants

The gene expression study was performed in 39 patients with biopsy-proven NAFLD (20 with SS and 19 with NASH) and 24 living liver donors (LD) as controls (S3 File).[18] Of those, 32 NAFLD patients (17 SS, 15 NASH) and 22 LD had plasma retinol levels measured and 14 SS, 12 NASH and 21 LD provided food records. Basic blood biochemistry and anthropometry were available for all participants (S4 File). The inclusion criteria for NAFLD patients were: male and female,18 years or older, alcohol consumption ≤20g/d; for NAFLD, a diagnostic liver biopsy and if known to have hyperlipidemia or diabetes, needed to be stable drug regimen. For LD, inclusion criteria were those of healthy living liver donors with a normal liver (no steatosis or cirrhosis) on imaging and/or histology. The exclusion criteria for LD and NAFLD groups were: any causes for liver disease other than NAFLD; regular intake of supplements (antioxidants, n-3 fish-oil, pre- or probiotics) or any experimental drug in the 6 months prior to study entry; pregnancy or breastfeeding; for NAFLD, anticipated need for liver transplantation within one year or complications of end stage liver disease such as variceal bleeding or ascites; concurrent medical illnesses or contraindications for biopsy; for LD, exclusion from liver donation. Liver tissue was collected during percutaneous needle biopsy for patients and during partial hepatectomy for liver donors. One portion was stored in 10% formalin for histology and another portion was stored in RNAlater (Qiagen, Hilden, Germany) for gene expression analysis (S2 File).

### Blood biochemistry analysis

Blood samples were obtained by venipuncture from all participants in the morning after an 8-hour fast. The samples for plasma retinol were centrifuged and stored frozen at −80°C until analysis. Standard blood biochemistry was measured at the Laboratory Medicine Program at the University Health Network. Fasting plasma glucose was measured by the enzymatic hexokinase method on an Architect c8000 System (Abbot Laboratories, Abbot Park, IL, USA), and serum insulin was assessed by radioimmunoassay (Immulite 2500, Siemens Diagnostics, Los Angeles, CA, USA). Insulin resistance was calculated from the fasting insulin and glucose values using the homeostasis model assessment of insulin resistance analysis (HOMA-IR).[29] Alanine aminotransferase (ALT), aspartate aminotransferase (AST), and alkaline phosphatase (ALP) in plasma as well as triacylglycerol, total cholesterol and high-density lipoprotein (HDL) cholesterol in serum were measured using the Architect c8000 system (Abbot Laboratories). Low density lipoprotein (LDL) was calculated as total–HDL cholesterol. Total bilirubin was measured with the diazol reaction. Plasma retinol levels were quantified by high-performance liquid chromatography (HPLC) (Varian Star, Agilent Technologies) equipped with a C18 column (Agilent Technologies, ZORBAX Eclipse Plus, 150 x 4.6 mm) for separation and a UV detector.[30] Retinol concentrations were measured at 325 nm. HPLC-grade hexane, ethanol and methanol; standard solutions retinol and retinyl acetate were purchased from Sigma-Aldrich (St. Louis, MO, USA). Tinted glass vials were used to protect the extracted samples from light.

### Assessment of liver histology

Samples were stained with hematoxylin and eosin for morphologic evaluation and Prussian blue to rule out iron loading. A single pathologist (SEF) reviewed the slides blinded. NASH was diagnosed per Brunt,[31] and the NAFLD activity score was calculated.[32]

### Hepatic gene expression analysis

The details of the gene expression analysis were described previously.[8] Briefly, total RNA was extracted using the *mir*Vana^TM^ miRNA Isolation kit (Life Technologies Corp., Carlsbad, CA, USA). The RNA concentration and purity were assessed with a Thermo Scientific’s NanoDrop 1000 Spectrophotometer (NanoDrop Technologies, Wilmington, DE, USA), and quality was checked spectrophotometrically with an Agilent 2100 Bioanalyzer (Agilent, Palo Alto, CA, USA). The Illumina Human HT-12 V4 BeadChip with the Whole Genome Gene–DASL HT Assay (Illumina Inc., San Diego, CA, USA), which covers >47,000 probes corresponding to 29,285 genes was used to examine hepatic gene expression.[18] Overall quality of the data was checked using R (v2.15.1) with the lumi Bioconductor package. During this step, nine outliers (4 NASH, 5 SS), were excluded from further analysis. Probes that did not show any signals were filtered and then only probes that were in the upper 80^th^ percentile of the distribution of intensities in at least 80% of the samples were retained. The three groups were compared using one-way ANOVA with Tukey’s post-hoc test, applying the Benjamini-Hochberg false-discovery rate (FDR) q<0.05 method to account for multiple comparisons. For the present study, a cut-off for up- or downregulation between the groups was not applied.

The following genes relevant to the aim of the present study were selected for further analysis: for retinol metabolism, *AKR1B10* (aldo-keto reductase family 1 member B10), *RBP4* (retinol binding protein 4), *RBP1* (cellular retinol binding protein 1), *ADH1A* (alcohol dehydrogenase 1), *ADH1B* (alcohol dehydrogenase 1B), *ADH1C* (alcohol dehydrogenase 1C), *DHRS3* (dehydrogenase/reductase (SDR family) member 3), *DHRS4* (dehydrogenase/reductase (SDR family) member 4), *RDH5* (retinol dehydrogenase 5), *RDH10* (retinol dehydrogenase 10), *RDH11* (retinol dehydrogenase 11), *ALDH1A1* (retinaldehyde dehydrogenase 1 family, member A1), *ALDH1A2* (retinaldehyde dehydrogenase 1 family, member A2), *ALDH1A3* (retinaldehyde dehydrogenase 1 family, member A3), *CRABP1* (cellular retinoic acid binding protein 1), *CRABP2* (cellular retinoic acid binding protein 2), *CYP26A1* (cytochrome P450, family 26, subfamily A, member 1), *CYP26B1* (cytochrome P450, family 26, subfamily B, member 1); and for transcription factors, *RARB* (retinoic acid receptor, beta), *RARG* (retinoic acid receptor, gamma), *RXRB* (retinoid X receptor, beta), *RXRG* (retinoid X receptor gamma), *PPARA* (peroxisome proliferator-activated receptor alpha), *PPARG* (peroxisome proliferator-activated receptor gamma) (S5 File). Gene expression data are publicly available from the NCBI Gene Expression Omnibus (GEO), https://www.ncbi.nlm.nih.gov/geo/, Accession No: GSE89632.

### Nutritional and dietary assessment

Weight and height to calculate BMI and waist circumference were measured. Participants were asked to complete a 7-day food record, estimating portion sizes with the 2D Food Portion Visual chart (Nutrition Consulting Enterprises, Framingham, MA). Nutrient intake was calculated using Food Processor SQL (ESHA Research, Salem, OR).

### Sample size calculations

The cross-sectional study is additional analysis based on the patient sample from a larger study that evaluated hepatic fatty acid composition and gene expression; sample size calculation was done for the main outcome in that study (PUFA composition), and the results of that study have been published (18).

### Statistical analyses

Data are presented as mean ± SD, median (interquartile range), or n (%) of patients as appropriate. For continuous variables, ANOVA with Tukey post-hoc test or Kruskal-Wallis H test and Mann-Whitney U test were used to determine differences among the groups, depending on the variable distribution. To analyze associations between variables, the Spearman rank order correlation coefficient was used. All tests were two-sided and performed at the 5% significance (alpha) level. The hepatic gene expression data were normalized using a quantile normalization followed by a “per probe” median centered normalization and log2 transformed for analysis. However, for correlation analysis, non-median centered gene expression data were used. The statistical analysis was performed using IBM SPSS Statistics version 22.0 and SAS 9.4.

To summarize expression patterns of genes related to retinoid metabolism we used a principal component analysis (PCA). PCA is a useful mathematical algorithm to assess similarities and differences between samples and determine whether samples can be grouped. [33] The PCA was based on 10 differentially expressed genes (*RXRB*, *RDH10*, *ADH1B*, *ALDH1A3*, *PPARA*, *CYP26A1*, *ALDH1A2*, *AKR1B10*, *DHRS4*, *RDH11*).

## Results

### Clinical and biochemical characteristics of NAFLD patients and liver donors

Age, gender distribution and alcohol intake were not different among the groups, but the proportion of smokers was higher in NASH compared to liver donors (Table 1). Patients with NASH had higher BMI than LD, but waist circumference was not different. Fasting insulin levels in patients with NASH were higher than SS and LD, and higher in SS versus LD, resulting in enhancement in HOMA-IR in NASH and SS vs LD. AST levels were higher in NASH compared to SS and LD, whereas ALT in patients with NASH was higher than SS and LD and in SS versus LD. Serum triacylglycerols were higher in the SS and NASH than LD. In accordance with the definition of the groups, NAFLD activity score was zero in LD and increased successively through SS to NASH.

**Table 1:** Clinical and biochemical parameters in liver donors and patients with simple steatosis and non-alcoholic steatohepatitis.

	n	LD	n	SS	n	NASH
**Age (years)**	22	37.4 ± 10.6	17	42.6 ± 13.9	15	44.4 ± 8.3
**Female sex (% (n/n))**	22	59 (13/9)	17	29 (5/12)	15	47 (7/8)
**Smoking (% (n/n))**	22	0.0% (0/0)	17	6% (1/16)	15	20% (3/12) ^a^
**Alcohol (g/d)**	22	0.0 (2.2)	17	0.39 (3.0)	15	1.0 (4.0)
**BMI (kg/m**^**2**^**)**	22	25.8 ± 4.2	17	28.5 ± 4.3	15	32.0 ± 5.9^A^
**Waist (cm)**	21	84.5 ± 21.7	17	99.3 ± 9.5	13	90.5 ± 38.2
**AST (U/L)**	22	20.3 ± 6.0	17	28.4 ± 5.9	15	54.5 ± 5.0^A^^,^^B^
**ALT (U/L)**	22	19.5 ± 11.4	17	51.1 ± 18.5 ^A^	15	85.1 ± 40.2^A^^,^^B^
**ALP (U/L)**	22	66.6 ± 15.8	17	67.5 ± 19.5	15	76.7 ± 22.6
**Bilirubin (μmol/L)**	19	10.3 ± 5.1	16	12.0 ± 7.4	15	11.3 ± 5.6
**Glucose (mmol/L)**	22	5.0 ± 0.6	15	5.0 ± 2.1	14	5.8 ± 3.3
**Insulin (pmol/L)**	18	21 (35)	16	62 (132)^A^	13	128 (62)^A^^,^^b^
**HOMA-IR**	18	0.87 (1.51)	15	2.8 (2.62)^a^	13	5.4 (7.53)^A^
**Total cholesterol (mmol/L)**	18	3.7 ± 2.0	16	4.8 ± 1.6	14	4.8 ± 1.7
**LDL (mmol/L)**	18	2.2 ± 1.4	14	2.7 ± 1.5	13	2.6 ± 1.3
**HDL (mmol/L)**	17	± 0.6	15	1.0 ± 0.44	14	1.1 ± 0.4
**Triacylglycerols (mmol/L)**	18	0.77 (0.57)	16	1.31 (1.35) ^a^	14	1.75 (1.38) ^A^
**Histology**						
**Steatosis (% of** **hepatocytes)**	15	0.0 (1)	17	40 (45)^A^	15	40 (35) ^A^
**Steatosis grading (% of patients (15) 0 / 1 / 2 / 3**	15	100/0/0/0 (15/0/0/0)	17	0/47/35/18 (0/8/6/3)	15	0/33/47/20 (0/5/7/3)
**Fibrosis stage (% of patients (n)) 0 / 1 / 2 / 3 / 4**	14	64/36/0/0/0 (9/5/0/0/0)	17	88/12/0/0/0 (15/2/0/0/0)	15	27/27/ 13/20/13 (4/4/2/3/2)
**NAFLD activity score (0–8)**	12	0.00 (0.00)	17	2.00 (1.00)^A^	15	4.00 (1.00) ^A^^,^^B^

LD: liver donors, SS: simple steatosis, NASH: non-alcoholic steatohepatitis, BMI: body mass index, AST: aspartate transaminase. ALT: alanine transaminase, ALP: alkaline phosphatase, HOMA-IR: homeostasis model of assessment for insulin resistance, LDL: low-density lipoprotein cholesterol, HDL: high-density lipoprotein cholesterol. Values given are mean ± SD, median (interquartile range), or percent of valid cases. Superscript letters show statistically significant difference from liver donors (a, A) and from SS (b,B).

a,b: p<0.05

A,B: p<0.01. ANOVA with Tukey’s post-hoc test was used for normally distributed data, and Kruskal-Wallis and Mann-Whitney U test for variables with skewed distribution.

### Plasma retinol

**Fig 2:**
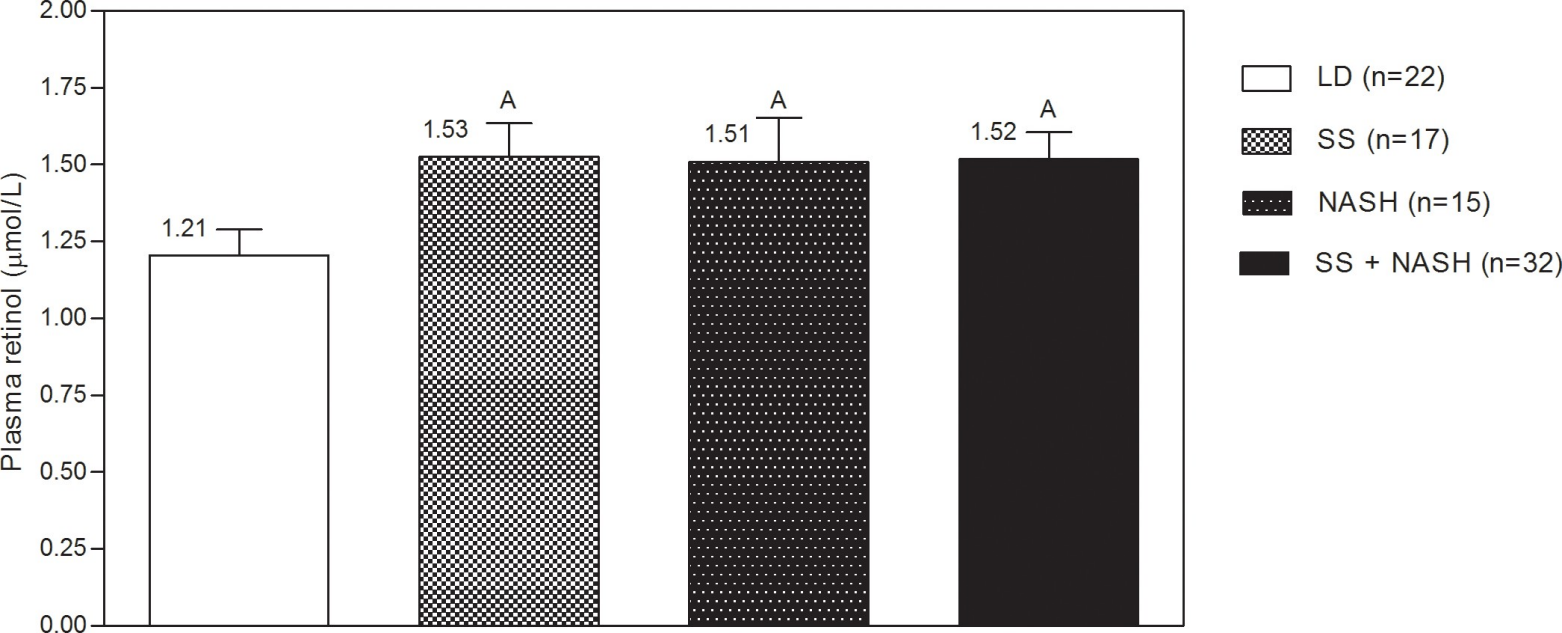
Plasma retinol levels in liver donors and patients with simple steatosis and non-alcoholic steatohepatitis. LD: liver donors, SS: simple steatosis, NASH: non-alcoholic steatohepatitis, NAFLD: non-alcoholic fatty liver disease (SS: simple steatosis + NASH: non-alcoholic steatohepatitis). A p<0.05, based on ANOVA with Tukey´s post-hoc test.

Plasma retinol concentrations were higher in NASH and SS group compared to healthy controls but did not differ between SS and NASH (Fig 2).

### Hepatic gene expression

**Fig 3:**
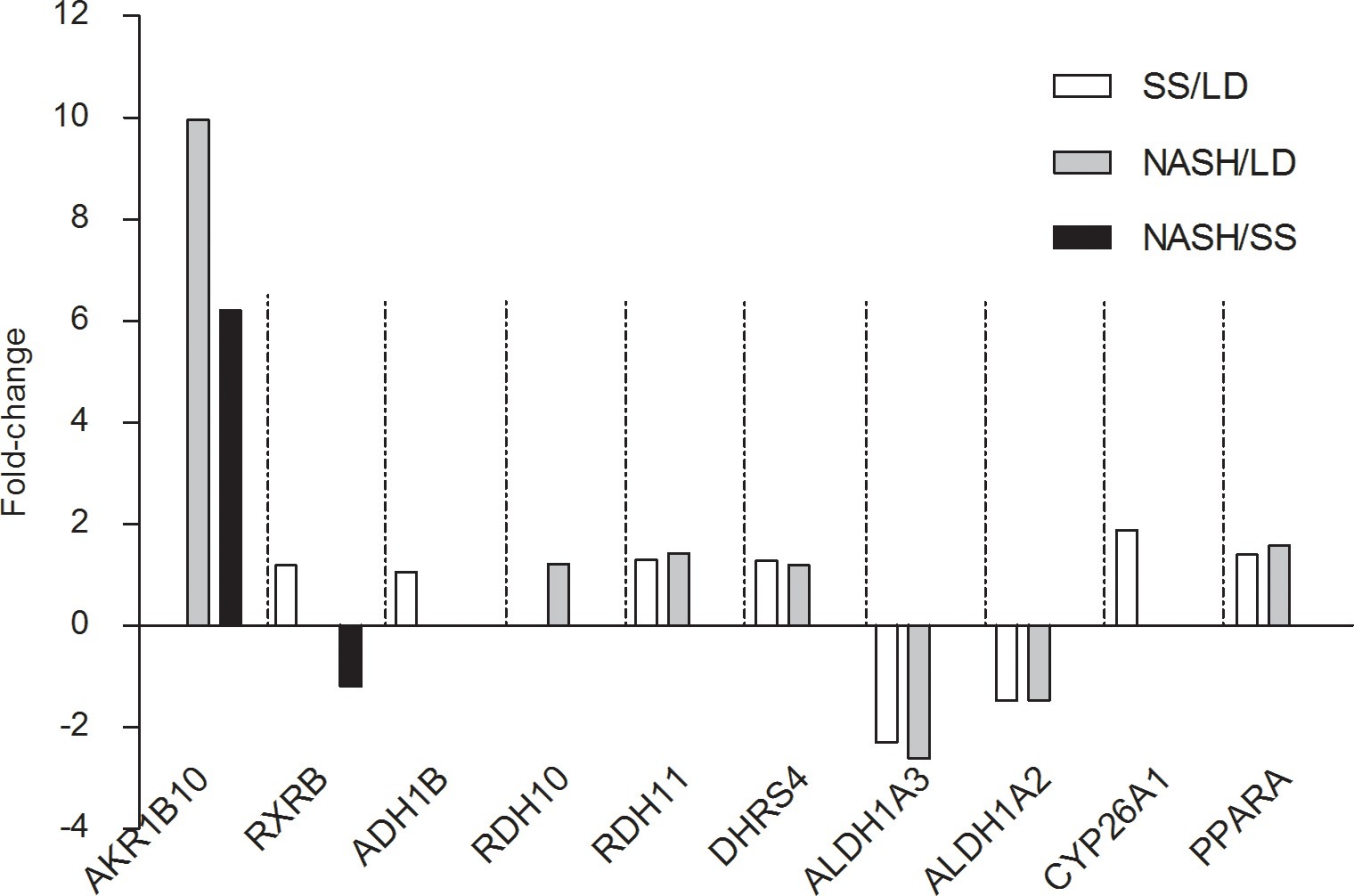
Genes related to retinol metabolism that were differentially expressed among liver donors and patients with simple steatosis or non-alcoholic steatohepatitis. Included are genes with a corrected post-hoc p-value <0.05. Gene expression levels are given as fold-changes between two groups. LD: liver donors, SS: simple steatosis, NASH: non-alcoholic steatohepatitis.

Patients with SS and NASH showed a different hepatic expression pattern of genes related to retinol metabolism compared to LD. Of the 24 relevant genes, 10 genes (8 for SS and 7 for NASH) were differentially expressed compared to LD (Fig 3). Only 2 genes (*AKR1B10* and *RXRB*) differed also between SS and NASH. The highest degree of overexpression was observed for *AKR1B10* in NASH versus both SS and LD. Four genes encoding for enzymes involved in retinal biosynthesis were also overexpressed: *DHRS4* and *RDH11* in SS and NASH compared to LD; *ADH1B* in SS versus LD, and *RDH10* in NASH versus LD. The transcriptional factor *PPARA* was overexpressed in both SS and NASH compared to LD, whereas *RXRB* was overexpressed only in SS vs. LD and underexpressed in NASH vs. SS. *CYP26A1* was overexpressed in SS vs. LD. Two genes encoding for enzymes involved in retinoic acid biosynthesis, *ALDH1A2*, and *ALDH1A3*, were underexpressed in both patient groups compared to LD. *RBP4* expression, which is important for the transport of retinol in blood, was not different among the three groups. In the NAFLD group, 4 patients with steatosis and 2 with NASH had diabetes. When excluding these patients with diabetes in a sensitivity analysis the results on hepatic gene expression remained unaltered (data not shown).

### Correlations between hepatic gene expression and plasma retinol levels

In the studied population, the enzymes *ALDH1A2* and *ALDH1A3*, responsible for retinoic acid synthesis from retinaldehyde, were inversely correlated with plasma retinol levels (Spearman´s rho = -0.41, p = 0.02 and rho = -0.30, p = 0.03, respectively). Furthermore, *RDH10*, *RDH11*, and *DHRS4*, all enzymes involved in maintaining an equilibrium between retinal and retinol, were positively correlated with plasma retinol levels (rho = 0.25, 0.25, and 0.25, respectively; all p = 0.03). *CYP26A1* (rho = 0.31; p = 0.01) and *PPARA* (rho = 0.30, p = 0.01) were also positively correlated with plasma retinol.

**Fig 4:**
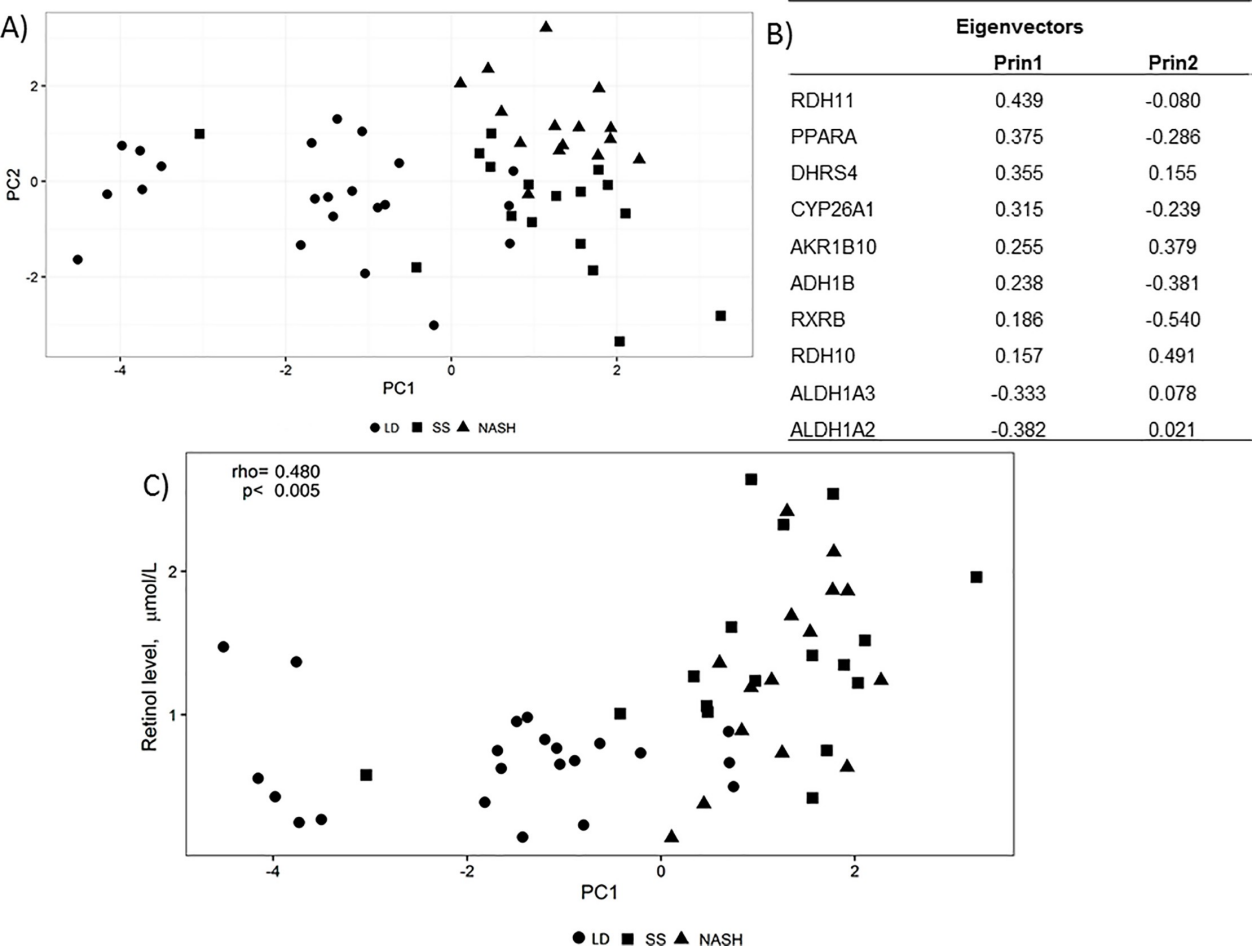
Principal component analysis including 10 genes related to retinol metabolism that were differentially expressed between patients with simple steatosis or non-alcoholic steatohepatitis and liver donors. (LD, closed circle; SS, closed square; NASH, closed triangle). Panel A shows the first two principal components extracted (PC1 and PC2). The location of each patient in the graph (PC1 versus PC2) separates patients with NAFLD (SS and NASH) from the LD group. This means that NAFLD patients have a distinct gene expression profile for retinol metabolism related genes compared to LD. In Panel B, eigenvectors are presented for PC1 and PC2. The 10 selected genes contributed similarly to PC1, except RXRB andRDH10 that had slightly lower eigenvector values. For PC2, RXRB, RDH10, ADH1B and AKR1B10 had the highest eigenvalues. Panel C shows the Spearman correlation between PC1 (separating NAFLD and LD) and retinol levels for all three groups combined. The analysis was repeated separately for patients and controls. In this case the correlation was only significant for NAFLD (rho = 0.480, p = 0.005) but not for LD.

The first principal component scores, which separated NAFLD and LD, were also significantly correlated with plasma retinol (Fig 4C).

### Dietary assessment

Based on food records, patients with SS consumed less energy, carbohydrates, and fat (g/d) compared to LD. NASH patients also reported lower carbohydrate intake than LD (Table 2). The intakes of vitamin A, carotene, β-carotene, and other macro- and micronutrients (data not shown) were not different.

**Table 2:** Food dietary intake in liver donors and patients with simple steatosis and non-alcoholic steatohepatitis.

Average Intake	LD (n = 21)	SS (n = 14)	NASH (n = 12)
	mean ± standard deviation		
**Energy (kcal/d)**	2,805 ± 1,191	1,781 ± 697^A^	2119 ± 506.8
	median (25th; 75th percentile)		
**Energy (kcal/d)**	2,665 (1937; 3394)	1,663 (1,105; 2,482)	2,083 (1,651; 2,380)
**Carbohydrates (g/d)**	347 ± 150.8	235 ± 83.5^a^	251 ± 78.1 ^a^
**Protein (g/d)**	111.7 ± 46.2	78.8 ± 34.6	103.8 ± 27.9
**Fat (g/d)**	111.3 ± 61.4	61.0 ± 30.5^A^	81.0 ± 27.8
**Carbohydrates (%)**	50.1 ± 7.3	53.6 ± 7.0	47.4 ± 8.2
**Protein (%)**	16.4 ± 4.0	17.7 ± 3.1	19.7 ± 3.7
**Fat (%)**	34.4 ± 7.8	30.2 ± 5.7	34.0 ± 6.3
**Vitamin A (IU)**	7,487 (11,753)	6,409 (9,938)	7,211 (15,419)
**Carotene (RE)**	576 (1,231)	528 (1,021)	634 (1,342)
**beta-carotene (μg)**	2,437 (6,175)	1,588 (3,662)	3,264 (7,956)

LD: liver donors, SS: simple steatosis, NASH: non-alcoholic steatohepatitis

Superscript letters show statistically significant difference from liver donors (a, A) and from SS (b,B).

a,b: p<0.05

A,B: p<0.01. ANOVA with Tukey’s post-hoc test was used for normally distributed data; Kruskal-Wallis and Mann-Whitney test were applied for non-parametric variables.

## Discussion

In the studied population, hepatic *AKR1B10* expression was highly upregulated in patients with NASH compared to LD and SS. In addition, the gene expression of several other enzymes participating in retinol metabolism was also dysregulated in NAFLD compared to LD, and the expression levels correlated with plasma retinol. To our knowledge, this is the first time these associations are reported in NAFLD patients.

The liver has a central role in the metabolism of retinoids, [34] which depends on several enzymes.[8,10] Hepatocytes are critically involved in the uptake and processing of dietary retinol into the liver, whereas non-hepatocytes cells like HSCs, play a central role in storing hepatic retinoid. [7] In NAFLD, disturbances of hepatic gene expression have been reported, [28,35] among them an increased expression of *AKR1B10* [17,18] and other genes involved in retinoid-metabolism (*ADH1A*, *ADH1B*, *ADH1C*, *RDH5*, *RDH10*, RDH*11*, *DHRS3*, *ALDH1A1*, and *ALDH1A3*) whereas *MYC* (v-myc avian myelocytomatosis viral oncogene homolog) was under-expressed. [28] *AKR1B10* overexpression is also considered a marker for HCC.[16,36] This is relevant, since retinoids can influence key processes like cell growth and differentiation, and consequently carcinogenesis and patients with NASH have increased risk for HCC. Until this report, no studies have assessed the expression of genes related to retinoid-metabolism and their association with plasma retinol in NAFLD patients.

Upregulation of *AKR1B10* in NAFLD could be due to the presence of oxidative stress,[37,38] particularly in NASH. [39] Aldehydes such as 4-hydroxynon-2-enal, 4-oxonon-2-enal, malondialdehyde and others oxidative stress-related compounds may induce *AKR1B10* as part of the cellular defense response against oxidative stress. [13,18,37] A pro-oxidant state is well documented in NAFLD patients compared to control subjects as evidenced by e.g., a higher content of protein carbonyls, GSH depletion, low catalase activity, an increment of both 3-nitrotyrosine immunoreactivity and production of O2•− and malondialdehyde by Kupffer cells.[39] However, in a previous study, done by our group, we did not detect any differences in oxidative stress (plasma and hepatic antioxidant power and liver lipids peroxides) between NAFLD and healthy controls.[18] This discrepancy may be due to different pro-/antioxidant parameters assessed in these studies.

High insulin levels and insulin resistance are present in NAFLD, especially in NASH, and this could also contribute to the increased *AKR1B10* expression.[40] This relationship was also suggested in our previous study[18] where HOMA-IR correlated positively with *AKR1B10* expression. Through this mechanism, insulin could play a role in disease progression from NASH to HCC. This relationship between insulin and HCC has also been suggested through animal models of NASH.[41]

No difference were found in dietary intake between groups, except for carbohydrates. However, food records should be interpreted with caution, especially in overweight and obese people, where underreporting has been suggested [19]. We also found that plasma retinol was higher in SS and NASH compared to LD despite similar dietary intake, which could be linked to an altered expression of several enzymes involved in retinol and retinal equilibrium. Higher plasma retinol was also reported by Bahcecioglu *et al*.[21] in patients with SS and NASH versus LD, but in that study, levels were also higher in SS than in NASH. In addition, several reports are available on RBP4, the specific carrier protein of retinol in the blood transporting retinol from storage sites in the liver to extrahepatic tissues.[42] Serum RBP4 correlates closely with serum retinol concentrations in obese and non-obese subjects,[43,44] and the use of serum retinol as a marker for retinol concentrations seems also appropriate for clinical populations.[43] An increase in plasma RBP4 levels has been observed in NAFLD patients diagnosed by ultrasound compared to controls [22,23] but no differences were found between SS and NASH.[24–26] In our study, *RBP4* expression was not different between patients and liver donors despite higher plasma retinol in NAFLD. This is consistent with results from Terra *et al*., who reported a lack of correlation between hepatic expression of *RBP4* and systemic levels in controls and in morbidly obese women with and without NAFLD.[26] In summary, these findings suggest that other causes may disrupt the relation between retinol and RBP4, or, alternatively, hepatic *RBP4* expression does not translate into serum RBP4 levels.

The changes in plasma retinol may be the result of an altered retinoid metabolism in NAFLD.[22] Retinol status has an important role in liver homeostasis. Several reports have suggested a link between retinol and its derivate retinoic acid in liver regeneration and pathogenesis including inflammation, steatosis, fibrosis, cirrhosis, and cancer.[16] Considering, that biosynthesis of retinoic acid is the only established function of retinol, apart from the synthesis of retinaldehyde in the eye, and also that each enzyme contribution is a key factor in retinoid metabolism,[45] we speculate that differentially hepatic gene expression of enzymes involved in retinol-retinal equilibrium (*AKR1B10*, *RDH10*, *RDH11*, *DHRS4*, *ADH1B*), alter plasma retinol levels.

This is important, as the increased conversion to retinol may potentially reduce hepatic retinaldehyde bioavailability, which in turn could lead to lower levels available to form retinoic acid,[46] therefore influencing retinoic acid signaling and carcinogenesis (Fig 1). This is consistent with lower hepatic retinol reserves in patients with NASH compared to SS reported recently.[27] As these patients have a high risk of HCC and approved treatments are missing, the underlying mechanisms warrant further investigation.

Other genes related to retinoid metabolism that were also differentially expressed between patients and controls deserve more research. *RDH10* was overexpressed in NASH patients compared to SS and LD. *RDH10* is a pivotal enzyme involved in retinaldehyde biosynthesis from retinol.[45] An overexpression of *RDH10* and other enzymes [28,35] that generate all-*trans* retinal from all-*trans* retinol in the biosynthetic pathway of retinoic acid, may suggests a response mechanism to overexpression of *AKR1B10*. The clinical significance is not clear, as an increased expression of *RDH10* has been identified as a marker of tumor progression in other cancers such as lung cancer.[47] In contrast, in hepatocarcinoma HepG2 cells—a model for HCC—an overexpression of *RDH10* induced a significant anti-proliferative response.[34] Our results also showed an underexpression of *ALDH1A2* and *ALDH1A3* in SS and NASH, compared to LD, which is contradictory to a previous report describing overexpression of *ALDH1A1* and *ALDH1A3* in patients with NAFLD versus controls.[28] This could be due to differences in the patient population, as our participants had higher BMI compared to the previous study. Differences in diagnostic criteria for NAFLD and in gene expression analysis methods could be other explanations. Following the oxidation of retinol to retinaldehyde, *ALDH1A1*, *ALDH1A2* and *ALDH1A3* are the enzymes responsible for the second and irreversible step in the oxidation of retinaldehyde to retinoic acid.[7] The downregulation of *ALDH1A1* and *ALDH1A3* in patients with SS and NASH compared to LD may reduce the amounts of retinoic acid in the liver and consequently alter interactions with RAR and RXR. This in turn could dysregulate the expression of genes involved in cell growth and differentiation, development, and homeostasis. In agreement with our results, Liu *et al*.[48] reported that serum concentrations of retinoic acid in patients with SS and NASH were significantly lower than in controls and were inversely correlated with hepatic steatosis and liver injury. Further studies in NAFLD patients are needed to assess retinaldehyde in the liver and determine its relation with *ALDH1A1* and ALDH1A3 expression as well as implications for the NAFLD pathogenesis.

This study has some limitations. First, a relatively small number of patients could be recruited due to the invasive nature of the liver biopsy. Second, this is a cross-sectional study and therefore a causal relationship between altered gene expression, plasma retinol, and the relation with NASH cannot be established. Third, due to the small amount of tissue available from the liver biopsies, an analysis of retinol and metabolites in the liver was not possible. We also did not measure other metabolites in the retinoic acid biosynthesis pathway in plasma. Nevertheless, our data can generate new hypotheses and support future research projects. Subsequent studies should measure hepatic retinol, retinaldehyde and retinoic acid to identify the association with the hepatic expression of key enzymes in their metabolism.

As *AKR1B10* shares 70% sequence identity with aldose reductase (*AKR1B1*), it would be interesting to assess the role of *AKR1B1* in retinoids metabolism in NAFLD patients.[15] *AKR1B1* has been implicated in the development of diabetic complications and might play a role in the glucido-lipidic metabolism and adipose tissue homeostasis.[49] Furthermore, animal models could be used to establish the role of *AKR1B10* overexpression in connection with an altered retinoid metabolism in the progression of NAFLD to HCC. Animal studies have already shown a connection between hepatic lipid metabolism and retinoid metabolism, as changes in the expression levels of several transcription factors (*PPAR-α*, *RXR-α*, *UCP-2*, *SREBP-1c*) and enzymes involved in lipid metabolism (liver-*CPT-1* and fatty acid synthase) have been observed after treatment with retinoids. [50,51]

## Conclusions

Hepatic *AKR1B10* is highly overexpressed in patients with NASH compared to SS and LD. This *AKR1B10* overexpression may reduce hepatic retinaldehyde levels, which in turn can decrease retinoic acid, favoring NASH progression to HCC. Moreover, patients with SS and NASH show differential expression of genes related to the metabolism of retinol, a process that could reinforce an altered retinoic acid biosynthesis.

The liver is the most important organ for the storage and metabolism of retinol, and it contains the enzymes for retinol metabolism. The gene expression of several enzymes participating in retinol metabolism was dysregulated in NAFLD compared to LD, and the expression levels correlated with plasma retinol. In addition, hepatic AKR1B10 expression was highly upregulated in patients with NASH compared to LD and SS. An *AKR1B10* overexpression accompanied by an underexpression of *ALDH1A2* and *ALDH1A3* may favor NASH progression to HCC. Moreover, patients with SS and NASH show differential expression of genes related to the metabolism of retinol, a process that could reinforce an altered retinoic acid biosynthesis.

## Supporting information

S1 File Study protocol. (PDF)

S2 File TREND checklist. This list summarizes the information provided regarding how this cross-sectional study was designed, analyzed and interpreted.
(PDF)

S3 File Materials and methods. (PDF)

S4 File Clinical, food records and non-median centered gene dataset. This is a tab file that provides a detailed description of clinical data, food records and non-median_centered gene data of the NAFLD patients and liver donors.
(XLSX)

S5 File Genes dataset. This is a tab file containing a listing of genes expression information, and comparision results between liver donor, and NAFLD patients.
(XLSX)
