# Supplementary material for: Altered hepatic genes related to retinol metabolism and plasma retinol in patients with non-alcoholic fatty liver disease
Source: PLoS One. 2018 Oct 31;13(10):e0205747. doi: 10.1371/journal.pone.0205747 (PMC6209208; doi:10.1371/journal.pone.0205747)
Supplement: S1 File — (PDF) [file pone.0205747.s001.pdf]

20. Research Proposal

Project Title: Non-alcoholic steatohepatitis (NASH) versus simple hepatic steatosis: Is there a difference in the nutritional factors influencing lipid perioxidation and inflammation?

INTRODUCTION:

Non-alcoholic fatty liver disease (NAFLD) is an increasingly recognized condition that may progress to end-stage liver disease. It refers to a wide spectrum of liver damage ranging from simple steatosis to steatohepatitis, advanced fibrosis and cirrhosis. While simple steatosis has a benign course, steato-hepatitis (NASH) can progress to fibrosis and cirrhosis. In NASH, the pathological picture resembles that of alcohol-induced liver injury, but it occurs in patients who do not abuse alcohol[1-5]. The diagnosis is usually suspected in persons with asymptomatic elevation of aminotransferase levels, radiologic findings of fatty liver, or unexplained persistent hepatomegaly. The clinical diagnosis and liver tests have a poor predictive value with respect to histologic involvement[6]. The diagnosis and the severity of liver damage can only be confirmed with a liver biopsy[7-18].

NAFLD is associated with obesity, diabetes and hyperlipidemia, with a variable degree of disordered fatty-acid metabolism and insulin resistance[18]. Fat accumulation in the liver increases oxidative stress. This may lead to increased production of cytokines like TNF-α and TGF-β which can contribute to the development of steatohepatitis, fibrosis and insulin resistance.

Changes in FA composition within the liver may also influence lipid metabolism and inflammation. Animal studies showed that a lower polyunsaturated FA (PUFA) in the liver, particularly the n-3 PUFA family of fatty acids (FA), predispose to liver steatosis (7-10). Depletion of PUFA favors lipid synthesis over oxidation and secretion (11-13). There is also evidence that n-3 PUFA have an effect on the transcription of antioxidant genes, inflammatory response and ROS production (23). One human study (24) looked at differences in hepatic gene expression by microarray in morbidly obese patients (mean BMI 50 kg/m^2)(29 NASH, 12 steatosis, 7 obese controls and 6 non-obese controls): 34 genes related to extracellular matrix remodeling, liver regeneration and apoptosis were differentially expressed in NASH patients. These changes in liver FA composition may be influenced by dietary intake of PUFA, high rate of lipid peroxidation or low Δ-6 desaturase activity. In humans, variations of the content of PUFA in plasma, erythrocytes and adipose tissue correlate with dietary intake (25-29) but no suteis reported on liver FA composition and diet in NASH. None of the NASH studies on FA composition had a true healthy control group for comparison and no other studies have published liver FA composition data and their possible relations with gene expression.

Weight loss, treatment of hyperlipidemia and hyperglycemia, and discontinuation of toxic drugs are the mainstays of therapy[19]. However, many patients lack known risk factors[12] and these efforts cure only a few [9,10,12, 20-22]. In fact, although there could be improvement in liver tests and steatosis after weight reduction[19,23-29], the degree of necroinflammation and fibrosis may worsen [23,24,26,27,30,31] and rapid weight reduction may even accelerate disease progression[23,24,30,32]. So far, no medications reduce or reverse liver damage independently of weight loss[18]. Seven small pilot studies lasting one year or less were reported to date using various compounds[18]. Two used vitamin E, a potent liposoluble antioxidant: one improved liver tests in obese children[33] and another, in adults[34], improved both liver tests and histology.

Based on this, in patients with elevated liver enzymes, we plan to compare those with SS, NASH and subjects with minimal findings on liver biopsy to healthy controls (HC) by enrolling healthy living donors from the Transplantation Program. We will measure hepatic FA composition and expression of genes involved in lipid metabolism. In addition, we will measure hepatic and plasma markers of oxidative stress, nutritional status (anthropometry, body composition and dietary intake) and IR.

Recent animal studies also suggest that the intestinal microflora (microbiota) (IM) composition might play a role in the development of NAFLD[222-226]. However, there are currently no human data available to assess the role of IM in NAFLD. ***We therefore want to include a pilot project comparing the microbiota composition of patients with SS, NASH or minimal findings to healthy controls.***

One way by which IM could lead to fatty liver is a reduction of choline bioavailability due to bacterial metabolism of choline. ***We therefore also want to measure serum free choline in all three groups.***

The objectives: To determine and compare healthy control liver donors to patients with elevated liver enzymes who were found to have simple steatosis, NASH or minimal findings on liver biopsy, liver lipid peroxides and TNF-α; liver immunohistochemistry for adducts of MDA, a product of lipid peroxidation (LP), α-smooth muscle actin (α-SMA), a marker of hepatic stellate cell activation; and transforming growth factor (TGF-β), a profibrogenic cytokine involved in fibrogenesis; microbiota composition. In addition, other measurements include: insulin resistance parameters such as HbA1c, blood lipid profile, blood glucose, insulin and c-peptide; plasma lipid peroxide, antioxidant vitamins and antioxidant power; red blood cell membrane and hepatic fatty acid profile (n-6 PUFA and n-6/n-3 ratio); nutritional indices such as 7 days food record and anthropometry; liver enzymes; subject demographics and medical history. 2. To detect differences in hepatic gene expression between patients with NAFLD (SS+NASH) and healthy controls (healthy liver donors) by using cDNA microarrays. 3. To determine if there is an association between hepatic n-3 PUFA content and gene expression between simple steatosis and NASH patients. 4. To compare intestinal microbiota composition and serum free choline in the three groups.

BACKGROUND AND SIGNIFICANCE:

Epidemiologic features

Risk Factors: NASH is associated with a number of metabolic conditions, surgical procedures and drug treatments [35,36]. However, ***most patients with NASH have central obesity, up to 75% are female [8,37] and many have diabetes mellitus, hypercholesterolemia or hypertriglyceridemia***. The reported prevalence of obesity in several series of patients with NASH varied between 30 and 100%[7,8,10,11,20,28,38-42]. Truncal obesity seems to be an important risk factor, even in patients with a normal BMI[43]. The prevalence of NAFLD increases by a factor of 4.6 in obese people with body mass index (BMI) of at least 30[44]. However, regardless of BMI, the presence of type 2 diabetes mellitus significantly increases the risk and severity of the disease[17,45]. In NASH, the prevalence of type 2 diabetes varies between 10 and 75% and the prevalence of hyperlipidemia is between 20 and 92 percent[7,8,10,11,20,28,38-42]. About half of patients with hyperlipidemia were found to have NAFLD on ultrasound examination[46].

NASH is also described in obese children[47,48] and has increased in frequency over the past 10 years. The largest pediatric series published to date (36 patients) is from the Hospital for Sick Children in Toronto[49]. Most patients were obese (114-192% ideal body weight) and 5

developed diabetes. Risk factors and pathogenesis are thought to be similar to NASH in adults[50].

Prevalence of NAFLD and NASH

NAFLD affects 10% to 24% of the general population in various countries. The prevalence increases to 57.5%[51] to 74%[24,44] in obese persons. NAFLD affects 2.6% of children[52] and 22.5%[52] to 52.8%[53] of obese children. NAFLD is the cause of asymptomatic elevation of aminotransferase levels in up to 90% of cases once other causes of liver disease are excluded[54]. More specifically, NASH is the histologic diagnosis in 7%-11% of patients undergoing liver biopsy in the United States and Canada[8,13]. It is often diagnosed after abnormal "routine tests"[19,20] and is the most common cause of abnormal liver tests in North American adults[55]. ***Recently an estimate was calculated from the known prevalence of obesity and type 2 diabetes mellitus [18] based on US data,*** where obesity affects 22.5% of people 20 years of age or older[56] and steatohepatitis affects about 3% of the lean population (<110% ideal body weight), 19% of the obese population and almost half of morbidly obese people[45,17]. ***For year 2000[5] , an estimated 30.1 million obese adults in the US may have steatosis and about 8.6 million may have NASH.*** The association of diabetes and obesity may pose an added risk: among the severely obese patients with diabetes, 100% were found to have steatosis, 50% steatohepatitis and 19% cirrhosis[58]. Based on these numbers, the prevalence of NAFLD in USA seems to be substantially greater than the 1.8% prevalence of hepatitis C virus infection[59]. ***Since the prevalence of obesity in Canada is getting close to the one in the US, with 30.5% of Canadians between the ages of 20-62 with a BMI $\geq$ 27 and 13.5% with BMI $\geq$ 30 [60], NASH is becoming an important Public Health issue even in Canada.***

Prognosis and Burden of the Illness

Whereas simple steatosis is a benign condition, NASH characterized by the combination of steatosis, infiltration of mononuclear cells or polymorphonuclear cells (or both) and hepatocyte ballooning and spotty necrosis may progress to cirrhosis[9,19]. A finding of fibrosis in NAFLD suggests more advanced and severe liver injury. In a total of 673 liver biopsies performed in NAFLD[3,5,10,12,14-17], some degree of fibrosis is found in up to 66% of patients at the time of diagnosis, whereas severe fibrosis (septal fibrosis or cirrhosis) is found in 25% and well-established cirrhosis is found in 14%.

No major prospective longitudinal study has been carried out to determine the prognosis of NASH. From existing case series, patients with simple steatosis have an excellent prognosis**.** No significant clinical or histological deterioration occurred in 40 patients with pure steatosis over a median of 11-year follow-up[20]. In patients with NASH[8-10,61-66], 15-50% developed significant ***fibrosis or cirrhosis.*** In these 299 patients, eight deaths from liver failure, two further non-fatal cases of liver failure and one transplantation (total 11 = 3.1%) are described. Propst et al[67] have reported a 67% 5-year survival and 59% 10-year survival in 30 patients with NASH, compared with 38% and 15% respectively in 65 patients with alcoholic steatohepatitis**.** ***In a more recent study[38], 36% of patients with NAFLD died after a mean follow-up of 8.3 years; liver-related diseases were the second most common cause of death, exceeded only by cancer. There was a trend toward more liver-related deaths among those with NASH, explained by a higher prevalence of cirrhosis[38]. A better understanding of the mechanisms involved should help identify cohorts who are at high risk.***

Mechanisms

The pathogenesis of NAFLD is poorly understood[45,68-73]. Apart from simple steatosis (the "first hit"), a "second hit" is required to induce necrosis, inflammation and fibrosis for the development of NASH[74]. Mechanisms associated with disordered fatty-acid metabolism and insulin resistance, abnormal cytokine production due to portal endotoxemia and bacterial over-growth, and oxidative stress due to lipid peroxidation (LP)[75] have been proposed. The most recent hypothesis is that progression from simple steatosis to steatohepatitis and to advanced fibrosis results from[74] first, insulin resistance leading to accumulation of fat within hepatocytes, and secondly LP[76] inducing cytokine production[18].

Role of Obesity, Diet, and Insulin Resistance (IR)

A net retention of lipids within hepatocytes, mostly in the form of triglycerides, is a prerequisite for the development of NAFLD. The primary metabolic abnormalities leading to lipid accumulation are not well understood, but they could consist of alterations in the pathways of uptake, synthesis, degradation, or secretion in hepatic lipid metabolism resulting from IR. IR is the most reproducible factor in the development of NAFLD[27]. The molecular pathogenesis of IR seems to be multifactorial and several molecular targets involved in the inhibition of insulin action have been identified[77-81]. IR leads to fat accumulation in hepatocytes by two main mechanisms: lipolysis, which increases circulating fatty acids (FA) and hyperinsulinemia. *Lipolysis*: The relative mobilizability of stored fats is increased by the degree of unsaturation at a given carbon chain length. The process of lipolysis delivers these non-esterified (unsaturated) FFA to the circulation[101]. This process provides appropriate amounts of circulating lipid fuel to fat-oxidizing tissues. However, excess FFA availability can also induce IR, hypertriglyceridemia, and changes in hepatic insulin clearance[101], abnormalities often associated with NAFLD**.** The type of fat can influence lipid metabolism (83-88). N-3 PUFA intake can reduce fat accumulation compared to saturated fat and increase lipid mobilization in visceral adipose tissue (89). Several human studies (80-82, 90-93) have also shown that saturated fat is significantly associated with worsening of IR, independent of body fat, while monounsaturated and PUFA improves IR.

Excess delivery of unsaturated FFA to the liver also predisposes to liver steatosis and provides substrates for LP[90]. In fact, one study suggested that long-chain and very-long chain (unsaturated) FFA may be the important factor in the pathogenesis of NASH[81a]. *Hyperinsulinemia:* Steatosis also occurs when the amount of FA supplied to the liver exceeds the amount needed for mitochondrial oxidation, synthesis of phospholipids and synthesis of cholesterol esters. This is the presumed mechanism for steatosis in the setting of obesity[82], diabetes mellitus[83], excessive dietary intake of fats and carbohydrates, acute starvation, total parenteral nurition[84] and protein-calorie malnutrition[85]. Hyperinsulinemia and IR are key components in this. High levels of insulin block mitochondrial fatty acid oxidation, leading to accumulation of triglycerides and FA in the liver[86]. Increased concentrations of intracellular FA may be directly toxic to hepatocytes or lead to oxidative stress [87]. FA are also substrates and inducers of the microsomal lipoxygenases cytochrome P-450 2E1 and 4A[88,89]. The level of cytochrome P-450 2E1 is invariably increased in the liver of NASH and may result in the production of reactive oxygen species (ROS) capable of inducing LP of hepatocyte membranes[88]. Hyperinsulinemia also increases the synthesis of FA in hepatocytes by increasing glycolysis and favors the accumulation of triglycerides in hepatocytes by decreasing hepatic production of apolipoprotein B-100[18].

The role of IR and oxidative stress in NAFLD is also supportd by a recent human study[90] in about 10 subjects showing that NASH and simple steatosis were both associated with IR, even in the absence of diabetes and that there was a greater degree of IR and oxidative stress in the liver of patients with NASH compared with simple steatosis and normal controls. In addition, oxidative stress may even further contribute to IR by inducing cytokine production[73,91-93]. In fact, a direct link between TNFα and obesity-linked IR was established [26,94] and a correlation was demonstrated between the degree of adiposity, increased TNFα production and insulin resistance [95,96]. Some even suggest that TNFα[95] may contribute to obesity-related NAFLD.

Finally, the distribution of body fat is also important in the development of hepatic steatosis. One study has shown a significant correlation between the degree of hepatic steatosis and the waist-to-hip ratio[97] pointing to the importance of intra-abdominal or visceral fat as predictor of fatty liver[83,97,98]. Body fat distribution modulates fatty acid metabolism[99] and upper-body obesity is associated with an increased risk of IR, hyperlipidemia, NIDDM[100] and with increased basal FFA flux relative to lean tissue mass[101]. FFA are mobilized more rapidly from central (visceral) than from subcutaneous fat and drain directly to the liver via the portal vein[102].

Therefore NAFLD is associated with hyperinsulinemia and IR. This favors accumulation of FFA in the liver by increasing peripheral lipolysis (mostly unsaturated fatty acids) and hepatic uptake. Accumulation of fat in hepatocytes predispose to lipid peroxidation and oxidative stress. This may in turn increase production of cytokines and further contribute to IR.

Oxidative Stress and Lipid Peroxidation (LP)

Oxidative stress is an imbalance between the production of ROS from LP and the antioxidant defense status, leading to oxidative damage and inflammation of surrounding tissue. The main substrate for LP is unsaturated fat.

Evidence for oxidative stress. Obesity and fatty liver are associated with oxidative stress. Obese animal models showed increased LP products in plasma, LDL fraction, liver and/or urine[103-105]. Increased liver LP and breath ethane were also reported[72]. Acute or chronic fat deposition in the liver, regardless of cause, was associated with LP and the degree of LP increased with the severity of steatosis[72]. There are only a few human studies. Obesity was recently associated with increased ROS generation by leukocytes and increased indices of oxidative damage to lipids and proteins, when compared with normal subjects[106]. A decrease in these indices was reported after dietary restriction and weight loss[106]. Leptin, increased in obesity and NIDDM[107-110], was also associated with oxidative stress in endothelial cells and could activate NF-kB in an oxidant-manner [111]. One study[90] showed an increase in oxidative stress in the liver of NASH patients.

Obesity is also associated with lower levels of serum antioxidants including vitamin E[112-114], beta-carotene[112] and antioxidant enzymes such as catalase, superoxide dismutase and glutathione peroxidase[115]. Others showed a decrease in antioxidant levels in obese adults[116] and in obese children[117,118]. Alpha-tocopherol, alpha-tocopherol/circulating lipid, and beta-carotene were significantly reduced in obese children[112]. Antioxidant levels were determined in 4771 of the 6139 children participating in the NHANES III, 726 were obese. Plasma antioxidant levels (beta-carotene and alpha-tocopherol) were significantly lower in obese children regardless of age or gender[117]. Obese children did not differ from normal-weight peers in dietary recall of intake of foods rich in vitamin E or beta-carotene. This may suggest an increased consumption of antioxidants to scavenge ROS produced from increased LP. Chronic LP, in turn, may exhaust the antioxidant system and lead to reduction in antioxidant enzymes as well as antioxidant vitamins.

<u>Source of ROS</u>. Macrovesicular steatosis arises from the increased mobilization and availability of FFA, increased hepatic synthesis of FFA, increased esterification of FFA into triglyceride, and decreased export of triglyceride from the liver[37]. Microvesicular steatosis is associated with impaired mitochondrial beta-oxidation[37]. Regardless of the cause, steatosis will increase LP because of the main substrate, unsaturated FA. The higher the degree of unsaturation, the higher will be the risk of LP, because double-bonds are easily auto-oxidizable. The increased concentration of FA within the liver can also saturate mitochondrial beta-oxidation, leading to more oxidative stress. Hydrogen peroxide produced from LP can then be converted to highly reactive hydroxyl radicals (ROS) in the presence of free iron[119], leading to more damage especially if there is excess liver iron[5]. Another source of oxidative stress is cytochrome P450 CYP2E1[120], often upregulated in diabetes, obesity and NASH[88] and normally suppressed by insulin[121]. This may initiate further LP[122,123]. Induction of CYP2E1 probably is not the only cause of LP and oxidative stress in NASH and other pathways are likely implicated[88,89,124,125].

<u>Effect of oxidative stress.</u> One end product of LP, malondialdehyde (MDA), can activate hepatic stellate cells, stimulating collagen production and hence fibrosis[37]. LP is an important mechanism of hepatic stellate cell activation in vitro, resulting in increased pro-collagen-α1 gene expression[126-128]. The association between LP and hepatic fibrosis has been shown for a variety of liver diseases[129-133]. MDA may also cross-link cytokeratins to form Mallory bodies. Another end product, 4-hydroxynonenal, is strongly chemoattractant for neutrophils[37]. MDA and ROS may also activate NF-kB and cytokine production including TNFα and IL-8[73,91-93]. Cytokines like IL-8[134] elicit neutrophil chemotaxis. Cytokines also mediate hepatic inflammation, apoptosis, cholestasis and fibrosis[135-139]. ROS also induces the formation of transforming growth factor beta (TGF-beta). TNF-alpha and TGF-beta cause caspase activation and hepatocyte death[140,141]. In addition, TGF-beta activates collagen synthesis by stellate cells[142] increasing fibrosis. It also activates tissue transglutaminase, which cross-links cytoskeletal proteins, promoting the formation of Mallory's hyaline.

NASH is also associated with mitochondrial abnormalities[90]. ROS can alter mitochondrial DNA[143] and react with mitochondrial proteins to inhibit the transfer of electrons along the respiratory chain[144]. Mitochondria produce then more ROS, and this may promote hepatocyte death[145]. In that study, mitochondria had a 50% reduction in cytochrome c content and produced ROS such as superoxide anion at a greater rate. Mitochondrial hydrogen peroxide generation was also increased by 200% and the activities of antioxidant enzymes (catalase, glutathione peroxidase, superoxide dismutase) were abnormal. ROS-mediated release of TNFα by hepatocytes, Kupffer cells and adipose tissue[25] also impaired mitochondrial respiration and depleted mitochondrial cytochrome c[146]. Mitochondrial ROS promote progression from steatosis to steatohepatitis and fibrosis by LP, cytokine induction and Fas ligand induction[18]. It plays a pivotal part in TNF-induced cell death[147,148] by causing expression of the Fas ligand in hepatocytes, which normally express the membrane receptor Fas [149].

<u>Role of antioxidants</u>. Oxidative stress is associated with lower antioxidant status. ROS consume antioxidant enzymes[150,151], glutathione[152] and vitamin E[112]. In turn, depletion of antioxidants hampers ROS inactivation, leading to further LP. Plasma total antioxidant status[104], plasma vitamin E/triglyceride ratio[153], and antioxidants in fatty livers such as glutathione, tocopherol and catalase[154] are lower in obese animals. Antioxidant levels in the liver could be corrected with vitamin E and C supplementation[154] and was associated with improved survival. Vitamin E also reduced LP and even reversed glucose-stimulated hyperinsulinemia[105]. In rat models of liver steatosis, treatment with various antioxidants decreased liver damage[155-157,120].

The effect of antioxidant in reducing inflammation is supported by in vitro studies where it reduced inflammation by inhibiting NF-kB activation[158-160]. Vitamin E prevented NF-kB activation[161] and reduced cytokine production and adhesion molecule expression[162-168]. Vitamin E inhibited TNFα induced NF-kB activation in human Jurkat T-cells[159]. Vitamin E also inhibited IL-1-mediated NF-kB activation in Caco-2 cell line[169]. Glutathione disulfide, another antioxidant, also inhibited the binding of activated NF-kB to its cognate DNA site[170]. In humans, vitamin E was reported to decrease monocyte function[171] and decrease production of TNFα[172]. In humans, two pilot studies looked at the effect of antioxidant in patients with NASH. One was an open-label study in 11 obese children (mean BMI 32.8, mean age 12.4 y). Vitamin E, between 400 and 1200 IU per day for 4 to 10 months normalized AST, ALT and ALP [33]. No liver biopsies were done. Another open-label study[34] with vitamin E 300 IU/day over 1 year was performed in 12 adults (BMI 29±1) with NASH diagnosed on liver biopsy. All patients had an impaired glucose tolerance test and were hyperlipidemic. Over 6 months prior to the vitamin E supplementation, there were no significant changes in liver enzymes with dietary therapy despite significant weight loss. After 1 year of vitamin E supplementation 9 of the 12 patients had a second liver biopsy reviewed "blindly". In 6 NASH patients, steatosis was improved, and, in five, inflammation and fibrosis were improved. None of the NASH patients had histological worsening after vitamin E and none experienced any side effects from the supplementation or the liver biopsy procedure. Plasma TGF-beta was also measured. It remained unchanged with the dietary therapy but it was significantly reduced after the vitamin E treatment.

Role of PUFA: PUFA may be a major factor involved in the mechanism of liver steatosis (134). PUFA, particularly those of the n-3 family can play pivotal roles as "fuel partitioners" (14). This is achieved by long-chain PUFA (20:4n-6, 20:5n-3 and 22:6n-3) that are able 1) to direct FA away from triacylglycerol storage, enhancing their oxidation, and 2) to direct glucose away from FA synthesis, increasing its flux to glycogen (12,13, 135). This so-called fuel partitioning action of PUFA requires both an adequate dietary supply of the 18:2n-6 and 18:3n-3 precursor FAs and an optimal activity of the delta-5/delta-6 desaturases to synthesize longer chain PUFA in the liver (12,13,135,136).

PUFA can also exert their beneficial effects by up-regulating the expression of genes encoding proteins involved in FA oxidation while simultaneously down-regulating genes involved in lipid synthesis. PUFA govern oxidative gene expression by activating the transcription factor PPAR-alpha (13,17,135). This will lead to the increased secretion of apolipoprotein B-100 and VLDL formation (18). Several animal studies showed that n-3 PUFAs can prevent and reverse steatosis (7-10). One mechanism of action of n-3 PUFA is activation of PPAR-α and PPAR-γ, which in turn increases the production of various enzymes that break down fat (84,85,139). Fish oil also inhibits a number of lipogenic enzymes (84,85,140).

Data in humans are very limited. One study suggests that regulation of PPAR- α genes in human hepatocytes may be different than in rodent hepatocytes (144). There are no human studies in NASH that have investigated the association between liver FA composition and gene expression. Recent data generated with cDNA microarrays in animals confirm the effects of n-4 PUFA on regulation of lipolytic and lipogenic gene expression as well as expression of genes involved in pathways such as oxidative stress response and antioxidant capacity, cell proliferation, cell growth and apoptosis, cell signaling and cell transduction (141).

There is also strong evidence that fish oil, containing n-3 PUFA, has a greater effect than safflower oil, containing n-6 PUFA, on the transcription of antioxidant genes, inflammatory response and ROS production (23). One microarray study showed that hepatic NF-kB gene expression was downregulated by the n-3 PUFA, docosahexaenoic acid (DHA) (142). Unexpectedly, cDNA microarray studies also showed that n-3 PUFA also modulated the expression of genes controlling cell proliferation, cell growth or apoptosis in normal tissues (23). Therefore from these cDNA studies in animals, it is clear that n-3 PUFA, provided in fish oil, modulates the expression of genes involved in lipid metabolism as well as other broad functions.
<u>*Other trials*</u>: Only a few open-label pilot studies with other compounds have been published in patients with NASH. Rosiglitazone[173] given in 30 subjects improved ALT and insulin sensitivity and reduced steatosis on CT imaging by week 24 (no liver biopsy). Metformin[174] given in 20 patients (500 mg tid x 4 months) improved insulin sensitivity and normalize ALT in 50% of patients[174]. Liver volume on imaging was reduced by 20% (no liver biopsy). Troglitazone[175] given in 10 patients (400 mg/d for $\leq$ 6 months) improved ALT in 7 but steatohepatitis persisted on liver biopsy in all patients. This drug was discontinued because of reports of severe hepatotoxicity[176,177]. Another study[178] evaluated ursodeoxycholic acid (UDCA) and clofibrate given for 1 year. UDCA may have a cytoprotective effect and clofibrate may decrease hepatic triglycerides. Twenty-four received UDCA (13-15mg/kg/d) and 16 with hypertriglyceridemia were placed on clofibrate (2g/d). Six withdrew because of side effects and 4 because of non-compliance. In UDCA group there was a significant decrease in ALT, AST, GGT and ALP as well as histologic grading of steatosis but no change in inflammation or fibrosis. Those on clofibrate showed no change in liver enzymes or histological grade of steatosis, inflammation or fibrosis[178]. Finally, betaine[179], a naturally occurring metabolite of choline, can raise S-adenosylmethionine levels that may protect against triglyceride deposition and hepatic steatosis. Ten patients received betaine (Cystadane) daily for 1 year. Seven completed the treatment. A significant improvement in ALT and AST occurred. There was an improvement in steatosis, necroinflammatory grade and stage of fibrosis after 1 year. Transitory GI side effects occurred in 4 patients. Serum triglyceride levels increased with betaine.

Microbiota Composition and NAFLD

The human gut contains at least 10^{13} microorganisms, collectively known as the microbiota. The microbiota is dominated by anaerobic bacteria and includes ≈500 – 1000 species.

<u>**Factors influencing the intestinal microbiota (IM)**</u>

IM can be influenced by a combination of factors ranging from mode of delivery to dietary habits and other host- and non-host associated factors [243,244] and is therefore characterized by high inter-individual variability.[245-248] For example, in regard to dietary habits, intake of cereal fiber, pectin, fat, or protein affects the ability of different bacterial groups to thrive in the gut.[249,250] Whereas high fat intake results in higher numbers of anaerobes, increased protein intake results in an enrichment of aerobic bacteria.[249] Other factors include previous gastrointestinal surgery, antibiotics, bacterial infection, drinking water purity, food quality, hygiene, climate, breastfeeding and exercise.[251,252] ***Therefore, IM can be influenced by several host and non-host factors, and diet seems to be one of the most important determinants.***[250,252]

<u>**Mechanisms linking IM and NAFLD**</u>

The following mechanism by which IM could influence obesity and related diseases are:

1) Increased energy extraction from food increasing energy provided to the host.[253-256]

2) Obesity, diabetes and NAFLD are metabolic disorders characterized by insulin resistance and low-grade systemic inflammation.[257,258] A growing body of evidence suggests bacterial lipopolysaccharide (LPS = endotoxin), as well as high-fat diet, are triggering factors for the onset of inflammation and insulin resistance. *Mechanism:* One important mediator of inflammation and insulin resistance appears to be Toll-like receptor 4 (TLR4), which can be activated by free fatty acids, especially saturated fatty acids, derived from high-fat diets.[259-262] Another trigger for TLR4 activation is LPS,[262,263] which could provide an explanation linking IM to obesity, insulin resistance, DM2, and NAFLD. Fecal butyrate, a short-chain fatty acid and a product of bacterial metabolism, is one of the factors involved in maintaining the integrity of the gut mucosa.[264] ***Therefore, changes in IM composition can promote translocation of LPS by increasing intestinal permeability, causing endotoxemia.***[253,259-261,263,265,266]

3) ***An additional mechanism linking IM to NAFLD is through bacterial choline metabolism***, which could reduce choline bioavailablilty. Choline is a dietary component essential for normal cell function [267]. In humans, dietary choline deficiency leads to fatty liver,[268,269] and liver damage due to inflammation and apoptosis.[267,270] The amount of choline and PC homeostasis could be influenced by IM, as the conversion of choline into methylamines by IM reduces the bioavailability of choline and mimics the effect of choline-deficient diets, causing NAFLD[271,272]. ***No human studies looked at the effect of IM on choline metabolism in NAFLD.***

Analysis of the Microbiome and Metagenome

Since the time of Pasteur and Koch, microbiologists have wanted to know three things about microorganisms: who is there; how many are there; and what are they doing?[28] The seminal work of Koch (eg. anthrax) and Pasteur (eg. cholera) developed the notion that a bacterium must be isolated as a pure clonal culture, and then inoculated into a healthy subject and only if the disease was reproduced could the cause and effect between microorganisms and phenotype (disease) be proven.[29] In the genomic age it is now possible to obtain a complete sequence of a microorganism so that the relationships of an ordered set of genes can be studied to provide insight into the structure and function of the proteome of the organism.[30] The genome sequence for Bacillus anthracis and Vibrio cholera have now been completed and sequences of multiple genomes (called comparative genomics) have provided important insight into the function of these organisms (called functional genomics).[31] But even Pasteur and Koch realized that it was not possible to isolate everything that could be seen under a microscope. It is known that less than 1% of microorganism in the biosphere can be cultured.[32] Further, it is well known that microorganisms do not exist as axenic cultures in nature and most diseases are the result of interactions between microorganisms.[33] For example, periodontal disease is the result of microbial biofilm activity in the periodontal pocket. Individuals with HIV infection often develop *Mycobacterium* infections.[34] ***Thus isolating an axenic culture by itself is not sufficient to understand how microorganisms interact in health and disease.***

With the advent of second generation sequencing technology (used in this grant) it is now possible to obtain the sequence of the genomes, or mixed genomes of a microbial community.[31] This area of study is called ***metagenomics***. Thus it is now possible to obtain the sequence of genomes within the phylogenetic context of the microbiome. The phylogenetic context is usually examined with conserved phylogenetic markers, the most common of which are 16S rDNA genes. For example in obese individuals (a focus of this grant) it appears as if the phylum Firmicutes is enriched. It is hypothesized that the Firmicutes have a higher percentage of polysaccharide degrading bacteria that are enriched in comparison to the Bacteroidetes.[35] The

importance of the Firmicutes is not the only relationship we will pursue but it illustrates how we will explore the functional relationships of the microbiome.

A more careful examination of the human gut microbiome reveals that the following microorganisms are the major polysaccharide degrading Firmicutes: *Roseburia intestinalis* (degrading xylan, starch), *Roseburia inulinivorans* (degrading inulin, starch), *Ruminococcus bromii* (starch), *Ruminococcus* sp. nov. (cellulose, xylan), *Eubacterium rectale* (starch) and *Bifidobacterium* spp.[36] However, the major Bacteroidetes that are also polysaccharide degraders are *Bacteroides thetaiotaomicron* (starch), *Bacteroides ovatus* (xylan, starch), and *Bacteroides cellulosilyticus* (cellulose). Thus, it is not plausible to explain obesity as simply an increase in Firmicutes vs. Bacteroidetes. It is more likely that there is a synergism between the Bacteroidetes and Firmicutes and that there is a relative enrichment of the polysaccharide degrades genes.[36] ***In the current project we will explore the FUNCTIONAL diversity of the microbiome***[37]***. This is an important distinction from a purely phylogenetic evaluation because we will evaluate the actual functional genes (eg. amylases, xylanases) to see if they are altered in patients with SS or NASH compared to healthy controls.*** Thus in this particular example we will bioinformatically identify genes associated with carbohydrate fermentation (glycosyl hydrolases) and assign them to phyla.[37] Utilizing this approach we will be able to explore potential relationships between disease phenotype and functional aspects of the microbiome and possible relationships with hepatic gene expression. In term of virulence we will explore the microbiome to identify specific virulence genes that are enriched in the gut. For example, bacterial lipopolysaccharide (LPS) is associated with obesity and insulin resistance. When we screen the metagenome we will generate for cell wall encoding genes that are important in microbial cell wall synthesis and outer membrane protein production.

Summary

NASH is associated with obesity, diabetes and hyperlipidemia. Fat accumulation in the liver is likely due to variable degrees of disordered fatty-acid metabolism and IR. Liver steatosis (unsaturated fat) increases LP and is associated with a reduction in the antioxidant defense system. This oxidative stress can lead to increased production of pro-inflammatory cytokines (TNF-α, TGF-β) contributing to the development of steatohepatitis and fibrosis. TNF-α may further contribute to IR. In addition, changes in FA composition within the liver may influence lipid metabolism and inflammation. In particular, n-3 PUFA have an effect on the insulin sensitivity, transcription of antioxidant genes, inflammatory response and ROS production. Microbiota composition might influence energy metabolism, and inflammatory tone and IR through increased endotoxemia and therefore could also play a role in the development of NAFLD.

HYPOTHESIS AND OBJECTIVES

Hypothesis 1: Patients with NASH are more oxidatively stressed compared to those with simple steatosis, minimal findings on biopsy and healthy control subjects with normal liver enzymes and liver histology. This is associated with 1) increased liver lipid peroxidation and cytokines (TNF-α and TGF-β), 2) increased unsaturated fat status (intake, tissue storage as measured in RBC, liver), and 3) reduced antioxidant status.

Hypothesis 2: There is a difference in hepatic gene expression activity between patients with NAFLD (SS+NASH) and healthy controls.

Hypothesis 3: Hepatic gene expression is altered in high or low n-3 PUFA content in liver total lipids of patients with non-alcoholic fatty liver disease (SS+NASH).

Hypothesis 4: Microbiota composition and the metagenome are different between patients with non-alcoholic fatty liver disease (SS+NASH) and healthy controls. This is associated with endotoxemia, changes in fecal butyrate and altered serum free choline and phospholipids in blood and liver.

Objective 1: To assess oxidative stress and nutritional status in patients with elevated liver enzymes who were found to have either simple steatosis or NASH or normal histological findings on liver biopsy by measuring liver lipid peroxides and TNF-alpha, liver pathology and immunohistochemistry (adducts of MDA, a product of lipid peroxides, alpha-smooth muscle actin (α-SMA), a marker of hepatic stellate cell activation; and transforming growth factor (TGF-β), a profibrogenic cytokine involved in fibrogenesis), AST, ALT, ALP, liver and red blood cell membrane fatty composition, IR parameters, plasma lipid peroxides, plasma antioxidant vitamins and antioxidant power, fasting glucose, HbA1c, lipid profile, subject demographics, medical history and medication use. In addition, nutritional data will include: (1) 7-day food record, variety and pattern; (2) anthropometry measurements. These measurements will be compared with healthy control subjects (healthy liver donors).

Objective 2: To detect differences in hepatic gene expression in patients with NAFLD (SS+NASH) and healthy controls using cDNA microarrays. Gene expression activity across the groups will be assessed by comparing average expression ratios of each group.

Objective 3: To determine in patients with non-alcoholic fatty liver disease (SS+NASH) whether there is an association between hepatic n-3 PUFA content and gene expression. For this, patients with low (n-3 PUFA $\leq$ 0.7%) versus higher PUFA (n-3 PUFA >0.7%) content will be compared.

Objective 4: To determine microbiota composition and intestinal microbiome (metagenome) in patients with NAFLD (SS+NASH) compared to healthy controls. We will also measure parameters related to IM, i.e. fecal butyrate concentration, endotoxin and bacterial DNA in plasma, serum free choline and PC/PE ratio in RBC and liver. An environmental questionnaire will assess possible host-factors influencing IM composition.

RESEARCH DESIGN AND METHODS

Subjects: Adult ambulatory subjects with suspected NAFLD, referred for a diagnostic liver biopsy because of altered transaminase and/or hepatomegaly, as well as healthy controls from the Living Donor Program for liver transplant recipients will be approached for the study. At the time of biopsy or surgery for healthy liver donors, a portion of the tissue will be snap-frozen in liquid nitrogen and stored in a –70 C freezer for measurement of hepatic gene expression, LP and fatty acid composition. Another portion will be fixed in 1% formaldehyde for routine histologic examination and immunohistochemistry staining by the Department of Pathology (Dr. M. Guindi). Other biochemical and nutritional parameters will then be measured.

Inclusion criteria:

a) Male and female patients, age >18 y

b) A liver biopsy with moderate to severe steatosis, either with absent or mild inflammatory infiltrate and/or fibrosis is defined as simple steatosis; moderate-gross macrovesicular fatty degeneration with inflammation (lobular or portal), with or without Mallory bodies, hepatocyte damage and/or fibrosis is defined as NASH[180-182] (see tables 1 and 2)

OR

No signs of steatosis, fibrosis or any other kind of liver disease on histology (minimal findings)

OR
For healthy control subjects, those with normal liver enzymes and normal liver imaging on ultrasound
c) convincing evidence of negligible alcohol consumption (<20g of ethanol per day) obtained from a detailed history, confirmed by at least one close relative; c) absence of any other possible cause for liver dysfunction.
Exclusion criteria:
a). Findings highly suggestive of liver disease of other etiology diagnosed as per routine medical investigation described under “general assessment”. (eg: chronic viral hepatitis, auto-immune chronic hepatitis, primary biliary cirrhosis and genetic liver diseases such as hemochromatosis, alpha-1 antitrypsine deficiency, Wilson’s disease and biliary obstruction).
b). anticipated need for liver transplantation in one year or complications of liver disease such as recurrent variceal bleeding, spontaneous portosystemic encephalopathy, resistant ascites or bacterial peritonitis;
c) concurrent medical illnesses contraindicating a liver biopsy: history of unexplained bleeding, hemophilia or abnormal coagulation results as per routine laboratory work-up or other reasons judged by the hepatologist to contraindicate a percutaneous liver biopsy.
d) chronic gastrointestinal diseases, previous gastrointestinal surgery modifying the anatomy, patients with diabetes requiring insulin.
e) medications known to precipitate steatohepatitis (corticosteroids, high dose estrogens, methotrexate, amiodarone, calcium channel blockers, spironolactone, sulfasalazine, naproxen or oxacillin) or regular intake of non-steroidal anti-inflammatory drugs, use of ursodeoxycholic acid or any experimental drug in the 6 months prior to entry.
f) regular intake of antioxidant vitamins, or omega-3/fish oil supplements, prebiotics, probiotics, antibiotics, or laxatives; in the 6 months prior to study entry
g) Pregnant or lactating

Procedures

General Assessment

All patients referred for abnormal liver enzymes will be assessed as per standard medical practice with thorough clinical history, physical examination and abdominal ultrasound. Liver disease of other etiology will be excluded. Standard laboratory investigation will be performed as medically indicated: total protein, albumin, aspartate transaminase (AST), alanine transaminase (ALT), γ-glutamyl-transferase (GGT), alkaline phosphatase (ALP), total serum bilirubin, hepatitis B and C serologies, antinuclear antibodies, anti-smooth muscle antibodies, anti-mitochondrial antibodies, iron levels, total iron binding capacities, ferritin levels, α-1 antitrypsin phenotypes and ceruloplasmin. When the timing is appropriate, the patient will undergo a percutaneous liver biopsy for diagnostic purposes. Healthy living donors are considered as control group for this study and are enrolled from the transplant clinic. Liver tissue will be obtained per-operatively at the time of the right hepatectomy (for healthy controls). Adequate tissue from a liver biopsy is found in almost all cases (99.8%) with a mean tissue length of 3.2 cm and overall complication rate requiring hospitalizations reported to be around 0.6%[183]. Performed by experienced hepatologist, complications are < 1/5000 and mortality < 1/12 000 [184].

After meeting the entry criteria and consenting to the study, baseline demographics and medical information will be recorded such as age, gender, medical history including diagnosis of diabetes and hyperlipidemia, alcohol intake, and drug regimen. If the patient is a smoker, the number of cigarettes smoked per day will be recorded at the same time as the food record. Instructions will be given on how to keep the food records and anthropometry measurements will be performed. Blood biochemistry will be done for AST, ALT, ALP, GGT, triglyceride, cholesterol, fasting glucose, HbA1c, lipid peroxides, antioxidant vitamins, antioxidant status and power and plasma/RBC fatty acid composition. C-peptide and insulin levels to assess IR (along with fasting glucose). ***These tests are standard or performed in Dr. Allard's laboratory (see appendix & Feasibility).***

Liver tissue assessment.

Pathology: Biopsy sections will be stained with hematoxylin and eosin for morphologic evaluation and Prussian Blue stain to rule out iron loading. ***These are standard techniques***. The histological evaluation will be performed by Dr. Guindi. *Immunohistochemistry:* This will be carried out using the formalin-fixed, paraffin-embedded sections, under Dr. M. Guindi's supervision. ***Immunochemistry is part of standard pathology laboratory techniques (see Feasibility).*** For adducts of *MDA, a product of LP*, monospecific antiserum to MDA-lysine adducts will be used as described previously[185-188]. For *α-SMA, a marker of hepatic stellate cell activation,* a mouse monoclonal anti-α-SMA antibody (clone 1A4;Sigma Chemical Company, St-Louis, MO, USA) will be used as previously described[189]. For *TGF-β, a profibrogenic cytokine involved in fibrogenesis*, sections will be stained with an anti-TGF-β1,2,3 monoclonal antibody (Genzyme Corporation, Cambridge, MA, USA) also previously described [190]. *TGF-β is also measured because, in addition to lipid peroxides, it may stimulate and sustain fibrogenesis by activating the lipocytes*[191]**.** For MDA, α-SMA and TGF-β staining, a validated semi-quantitative scoring system previously described will be used[192,193]. These techniques have been used in liver biopsies of patients with NASH with varying degrees of steatosis[192]. In this study, steatosis was associated with LP within zone 3 hepatocytes and with acinar inflammation. Alpha-SMA expression was associated with both LP and fibrosis.

Lipid peroxides in the liver: Liver biopsies will be stored at -70 C until analysis. Tissue samples will then be thawed, weighed and homogenized with ten fold (v/w) ice-cooled HCl-tris buffer at PH 7.4. Butylated hydroxytoluene (5mM) will be added to prevent ex-vivo lipid peroxidation. The suspension will then be centrifuged and the supernatant will be analysed using a commercially available kit (Oxis international, Portland, USA) which measures free MDA and 4-hydroxyalkenals. ***This technique is used in Dr. Allard's laboratory (see Feasibility, table 3).***

TNF-α in the liver: Hepatic tissue will be homogenized in 10 volume of 0.01 M PBS pH 7.4 for 1 minute in an ice-cold water bath. The homogenate will then be centrifuged at 10,000 g at 4 degree C for 30 min. The final supernatant will be analyzed for TNF-α by ELISA (R&D System Inc, Minn). ***This technique is used in Dr. Allard's laboratory (see Feasibility, table 3).***

Liver fatty acid composition: Liver fatty acids will be extracted into chloroform/methanol (2:1, vol/vol), containing BHT as an antioxidant and dried under nitrogen gas according to methods previously published and modified[212-214.] Fatty acid methyl esters will then be separated using a gas chromatograph equipped with a flame ionization detector and a 100-mm x 0.25 mm (0.2- um coating) SP2560 fused silica column. The temperature will be programmed as described previously[212,215]. Forty-three peaks will be identified from C12:0 to C 22:6 ω-3. Fatty acid composition was measured previously in our laboratory[216](see Appendix). This measurement also mirrors the fatty acid pattern of the diet over several weeks preceding the analysis[217,218]. In secondary analysis, results will be correlated with the markers of oxidative stress and gene expression.

Microarray / Gene expression

Portion of the liver biopsies (20-30 mg required) (193) will be immersed in RNAlater (Qiagen Mississauga, ON) and total RNA will be extracted by modified Trizol method as described previously (194). Two micrograms of total RNA from each biopsy specimen or from Universal Human Reference RNA (Stratagene, La Jolla, CA) will be amplified using the MessageAmp aRNA kit (Ambion, Austin, TX). Previous work from Dr. McGilvray lab indicated the gene expression profiles from amplified RNA have a good correlation with those developed from nonamplified RNA (194). Human single spot microarrays comprising 19 000 human clones will be used (UHN Microarray Center; http://www.microarray.ca/support/glists.html) which include genes involved in lipid metabolism including desaturases enzymes, oxidative stress, inflammation and fibrosis pathways and liver diseases (see examples in table 3). For each array, 5 ug of liver amplified RNA will be compared with 5 ug of amplified reference RNA. During reverse transcription, liver complementary DNA will be labeled with Cy5 and reference cDNA with Cy3 (193). Hybridization will be performed overnight at 37^{0}C (DIGEasy; Roche Diagnostics, GmbH, Mannheim, Germany). Arrays will be read by GenePix 4000A laser scanner and quantified with GenePix Pro software (Axon Instruments, Union City, CA). Microarray data will be normalized using an R-based, intensity-dependent LOWESS scatter plot smoother (http:// 142.150.56.35/~LiverArrayProject/home.html) (195-197).

In order to verify microarray data, two-step real time PCR will be performed after reverse transcription of 5 ug of amplified RNA with 5 ug pd(N)6-random hexamer primer (Amersham, Oakville, Ontario). The resulting complementary DNA will be used as a template for real-time PCR quantification with the Quantitect SYBR PCR Kit (Qiagen), and real time PCR (normalized to beta-actin) will be performed using the DNA Engine Opticon 2 cycler (MJ Research, Reno, NV). For designing gene-specific primers, Primer 3 on-line free software will be used (http://frodo.wi.mit.edu/cgi-bin/primer3/primer3.cgi/).

<u>Nutritional Assessment.</u>

<u>Anthropometry</u>. Weight and height will be measured to calculate BMI. Obesity is defined as BMI >30 kg/m^2, a cut-off which represents more than 120% of ideal body weight. Right upper arm circumference will be measured. Triceps, subscapular and supra-iliac (above the iliac crest) and waist skinfold (mm) will be measured to the nearest mm using Lange skinfold calipers. Waist to hip ratio will also be measured. From these measurements, mid-arm circumference, arm muscle area and total body fat will be calculated and expressed as percentiles of normal published values[194]. ***These anthropometric techniques were used previously by our group[195] (see appendix and feasibility, table 3).***

Food intake: Food records will be kept for 7 days to control for potential differences in ingestion of different levels of dietary antioxidants between the 2 groups and different fat intake, all this influencing markers of lipid peroxidation and possibly inflammation. For food records, the subjects eat their regular meals and itemize the fraction (to the nearest ¼) of portions of food consumed and record it on a standardized form collected by the nutritionist. The data will be analyzed using Minnesota Nutrition Data System, version 4.0 (Minneapolis, MN). Dietary variety within food groups and intake pattern will also be assessed according to the technique described[196]. ***We have used food record techniques in several of our studies including for micronutrients[197-201]. These techniques were found to be valid and reliable for use in clinical studies[202](see appendix).***

Oxidative stress assessment (used by Dr Allard, Appendix, Feasibility, Table 3).

Plasma lipid peroxides: Blood will be collected in EDTA-containing vacutainers and plasma will be separated by centrifuging at 2400 rpm for 10 min. Plasma lipid peroxides will be assessed using a commercially available kit (Oxis international, Portland, USA).

Plasma Antioxidant vitamins: Blood will be collected in EDTA-containing tube and centrifuged promptly. Plasma will be removed and frozen at -70 C for future assays. For vitamin C, plasma will be stabilized immediately with 100 g/L metaphosphoric acid (1:1) prior to freezing and later analysed by spectrophotometry[203]. Alpha- and gamma-tocopherol[204] and beta-carotene[205] will be analysed by HPLC and fluorescence spectrophotometry.

Plasma antioxidant power: This will be assessed by a commercially available kit (Oxis Research, Portland, USA)[206]. Assay is based upon the reduction of Cu++ to Cu+ by the combined action of all antioxidants present in the sample. A standard of known uric acid (a water soluble anti-oxidant) concentration is used to create a calibration curve. The results are then expressed either as "mM uric acid equivalents" or as "uM copper reducing equivalents"[207].

Fatty-acid and Insulin Resistance (IR) assessment (used by Dr. Allard, Feasibility, Table 3, Appendix)

Insulin resistance (IR): Insulin levels will be measured using an immunoenzymometric assay (MEIA, Abbott Diagnostis, IL). C-peptide will be measured using a double antibody competitive radioimmunoassay (Diagnostic Products Corp, CA). First, IR will be measured indirectly as described by Dixon[208] using the homeostasis model assessment (HOMA)[209, 210]. This is a validated method of estimating IR and beta-islet cell function using the fasting plasma glucose and C-peptide measures. Secondly, a quantitative insulin sensitivity check index (QUICKI)[211] based on the log transformed insulin-glucose product will be used. This index correlates well with the hyperinsulinemic euglycemic clamp and therefore its reverse is a good indirect measure of IR: IR index = {(log insulin) + (log fasting plasma glucose)} = 1/QUICKI. These methods showed significant differences in IR index, insulin sensitivity (HOMA%S) and beta cell function (HOMA%B) between NASH and simple steatosis[208].

Red blood cell and plasma fatty acid composition: Red cell membrane and plasma fatty acids will be extracted into chloroform/methanol (2:1, vol/vol), containing BHT as an antioxidant and dried under nitrogen gas according to methods previously published and modified[212-214.] The residue then dissolved in 0.1 mL chloroform is applied to silica gel thin-layer plates to separate the phospholipid, cholesterol, triglyceride and free fatty acid bands. Each band will then be scrapped and transmethylated with boron trifluoride-methanol at 90 C for 30 min. Fatty acid methyl esters will then be separated using a gas chromatograph equipped with a flame ionization detector and a 100-mm x 0.25 mm (0.2- um coating) SP2560 fused silica column. The temperature will be programmed as described previously[212,215]. Forty-three peaks will be identified from C12:0 to C 22:6 ω-3. Fatty acid composition was measured previously in our laboratory[216](see Appendix). This measurement also mirrors the fatty acid pattern of the diet over several weeks preceding the analysis[217,218].

Free choline in serum: Blood will be collected in serum tubes without anticoagulant. Tubes will be inverted to mix and put immediately on ice and then centrifuged 10 min, 2000g at 4ºC to separate serum. Aliquots (0.5 mL) will be stored at -80ºC until analysis, which will be done by our collaborator (K. da Costa, University of North Caroline, Chapel Hill). Choline will be extracted from serum by the method of Bligh and Dyer,[241] and separated and quantified directly using liquid chromatography/electrospray ionization-isotope dilution mass spectrometry (LC/ESI-IDMS) after addition of an internal standard labeled with stable isotopes to correct for recovery.[242]

Plasma endotoxin: Plasma samples will be diluted to 10% with endotoxin-free water and then were heated to 85°C for 12 min to inactivate inhibitory plasma proteins. Endotoxin concentration will be measured using a commercially available endpoint limulus amebocyte lysate assay (Charles River) for a concentration range of 0–1200 endotoxin units/L.[273]

Bacterial DNA in plasma: DNA will be extracted from 200 μL of plasma using the DNeasy Blood and Tissue Kit (Qiagen), according to the manufacturer's instructions. Quantitative polymerase chain reaction (PCR) for measurement of bacterial 16S rDNA will be performed.[274] Bacterial 16S rDNA in plasma will be measured in addition to endotoxin, as the commercially available kit for plasma LPS "has greater interassay variability than does the bacterial 16S rDNA assay (25% vs. 10%)".[274]

IM composition and short-chain fatty acids in stool samples (Drs. Allard, Comelli, Krause)

Samples will be collected at the time of liver biopsy or hepatectomy. At enrolment, participants receive a stool collection kit, including a plastic collection/storage container with a tightly closing lid, an insulated bag and cooling elements. They will collect one stool sample within 24 hours of the biopsy/hepatectomy appointment. The stool will be frozen immediately after defecation in the patient's home freezer. Patients will bring the frozen sample in the insulated bag with cooling elements to their appointment at the hospital, where it will stored at -80°C until analysis. This method of stool collection has been used in a study to establish a human gut microbial gene catalogue by metagenomic sequencing, which was published in Nature[187] and is acceptable to the patients.

Microbial composition and metagenome analysis: DNA will be extracted with standard methodology and checked for quality as described previously.[188] To obtain microbial composition data the V1 to V4 region of the 16S rDNA will be amplified with conserved barcoded primers.[189] In our laboratory (Krause) we have validated the use of 40 pairs of barcoded primers that represent a balance between obtaining adequate coverage and cost.

Sequences will subsequently be sorted using a custom Perl script and analyzed bioinformatically (see *Analysis Plan specifically for Intestinal Microbiota and related parameters*). Using multivariate and hypothesis testing approaches samples will be clustered into groups with the greatest similarity. We anticipate/budget that we will generate approximately 20 such groups. The metagenome will then be sequenced using pyrosequencing technology. Currently we (Krause lab) need approximately 4 Gbp of sequence to get reasonable coverage of a metagenome. Bioinformatic analysis will be as described below.

Quantification of microbial groups of special interest: Specific microbial groups that appear to be different between patients and controls according to the pyrosequencing results will be analyzed by real-time quantitative PCR. It is feasible to quantify 20 organisms and groups in approximately half of the samples by PCR. The selection will be based on the greatest differences on pyrosequencing. We will perform primer design based on the sequence data generated in the sequencing phase of the study. Groups that are expected to be of special interest are listed below:

Group	Reference	Group	Reference
Eubacteria (total counts)	Furet et al. [2]	Roseburia	Adapted from Belenguer et al.[191]
Firmicutes (phylum)	Adapted from Haakensen et al.[192]	Enterobacteriaceae	Adapted from Matsuda et al.[193]
Clostridia, cluster IV	Adapted from Song et al.[194]	Mollicutes *(Eubacterium)*	Adapted from Schwiertz et al.[195]
Clostridia, cluster XIV	Adapted from Song et al.[194]	Bacteroides/Prevotella	Furet et al. [2]
Clostridia, cluster IX	Adapted from Song et al.[194]	Archaea (*M. smithii*)	Adapted from Palmer et al. [196]
Lactobacilli	Haarman and Knol[197]	*F. prausnitzii* (species)	Adapted fr. Ramirez-Farias et al.[198]
Bifidobacteria	Furet et al.[2]		

We will use the extracted fecal DNA together with the TaqMan Gene Expression Master Mix and specific TaqMan primers-MGB (Minor Groove Binder) probe sets (Applied Biosystems). Primers are designed to amplify 16S rDNA for the following groups/genera of gut microorganisms: Firmicutes, Clostridia clusters IV, IX and XIV, Lactobacilli, Bifidobacteria, Roseburia, Enterobacteriaceae, Mollicutes, Bacteroides, Archaea, Eubacteria, and *F. prausnitzii*, as well as total counts. Primers and probe sets to be used are, or will be adapted from, those available in the literature (*Table 5*). Assays will be run in triplicate using a 7900HT thermocycler with a 384 wells block (Applied Biosystems). Number of cells of each microorganism in fecal samples will be calculated by interpolation from previously constructed standard curves (obtained from ten-fold serial dilutions of known quantities of bacterial DNA)[190] using the SDS software version 2.3 (Applied Biosystems). Numbers of bacteria will be expressed by g of feces (dry weight). These measurements will be performed in Dr. Comelli's lab.

Environmental Questionnaire: Participants will be asked to fill out a questionnaire to assess environmental factors that could influence IM, including: place of birth, ethnic/cultural background, breastfeeding as infant, allergies, gastrointestinal diseases and abdominal surgery, use of antibiotics or laxatives, consumption of pre- and probiotics, international travel, and pets. Food intake and physical activity, which might also influence IM composition are recorded separately.

Statistical analysis and sample size

Comparison among groups will be made using One-way ANOVA with Bonferroni corrections to control for multiple comparisons or Wilcoxon-Signed Rank test as appropriate. Proportions will be tested using Fisher's exact test. Pearson correlations will also be performed between various variables. A non-parametric version of a Welch's based t-test will also be used in examining differences between means of gene expressions on paired basis as follows SS/NASH, SS/healthy controls, NASH/healthy controls. In patients with non-alcoholic fatty liver disease (SS+NASH) a comparison of those with low hepatic n-3 PUFA ($\leq$ 0.7%) to those with high n-3 PUFA (> 0.7%) content will be made. Comparison will be performed by using unpaired t test or Wilcoxon test, as appropriate. Associations between hepatic FA composition, gene expressions and liver histology will also be determined using the R package 'globaltest'(213).
Sample size was estimated initially to be 50 patients with simple steatosis and 50 patients with NASH based on previous studies performed in this population[208,219,220]. Currently, 100 patients have consented and 38 were found to have NASH, 20 with simple steatosis and 17 with minimal findings on biopsy. Because gene expression was not done in these subjects, an additional 30 patients with SS and 30 with NASH is required for this study. Thirty healthy liver donors will also be included as healthy controls. A test will be considered statistically significant for those p-values<0.05. Statistics will be performed using SPSS package.

Analysis Plan specifically for Intestinal Microbiota and related parameters

(a) We will first examine all data visually via graphics/ plots along with a descriptive summary for each measurement variable in each experiment. Unusual data points such as values below the range limit or missing points will be re-examined. Univariate analysis will be performed for all measurement variables. Skewness and other departures from normality will be reviewed, and the variations across time points will also be examined.

Methods of statistical analysis to be used include analysis of variance (ANOVA), t-test, correlation analysis (e.g. Pearson's product-moment method), and multiple regression, where outcome variables are continuous. When using tests that depend on the normality assumption, such as t-test and F-test ANOVA, the validity of this assumption will be examined carefully. When group variances vary significantly across time points, data transformations (e.g. Box-Cox formula) will be applied prior to the analysis. In situations where departures from normality are evident, nonparametric methods (e.g. Kruskal-Wallis test, and Spearman's rank-order measures for correlation coefficients) will be used to summarize and analyze the data. When categorical/binary outcomes are considered, chi-square test, Fisher's exact test, and logistic regression (incl. proportional odds and continuation ratio models) will be employed. When multiple variables are considered simultaneously (e.g. befidobacteria in relation to duration of intervention and improvement of liver steatosis (yes/no)), main effects and interactions between factors will be tested via regression models.

For all analyses, the Type I error will be set to 5%, i.e. alpha=0.05, for all calculations of confidence intervals and for performing the various hypothesis-testing procedures. Model diagnostic tools and cross-validation methods[227] will be considered for model assessment. Statistical analyses will be carried out using the statistical software packages SAS Version 9.2, and R Version 2.11.

(b) Specifically, to assess the link between intestinal microbiome and disease, the following analyses will be performed:

To account for the correlations between measures taken from the same subject (at baseline, 6 months and 12 months), generalized linear mixed models, including the generalized estimating

equations approach, will be employed to examine the group difference over the 12-month intervention period in outcomes, such as the number of *Bifidobacteria* in stools and other IM compositions. Correlations between changes in IM and liver inflammation (via biopsies at months 0 and 12) will be investigated using the mixed model approach to incorporate variables such as age, gender, and BMI, etc. Where applicable, suitable variance-covariance structures will be explored, and model assessments will be determined by, for example, the Akaike Information Criteria (AIC) and other related criteria (SBC, CAIC).

Analysis Plan specifically for Intestinal Microbiota and related parameters

(a) We will first examine all data visually via graphics/ plots along with a descriptive summary for each measurement variable in each experiment. Unusual data points such as values below the range limit or missing points will be re-examined. Univariate analysis will be performed for all measurement variables within groups. Skewness and other departures from normality will be reviewed, and the variations across groups will also be examined. Hypotheses will be tested using the statistical methods given below.

Methods of statistical analysis to be used include analysis of variance (ANOVA), t-test, correlation analysis (e.g. Pearson's product-moment method), and multiple regression, where outcome variables are continuous (e.g. ratio of Firmicutes to Bacteroidetes (F/B), HbA1c). When using tests that depend on the normality assumption, such as t-test and F-test ANOVA, the validity of this assumption will be examined carefully. When group variances vary significantly across groups, data transformations (e.g. Box-Cox formula) will be applied prior to the analysis (such as ANOVA for AST in SS/NASH vs. controls). In situations where departures from normality are evident, nonparametric methods (e.g. Kruskal-Wallis test, and Spearman's rank-order measures) for correlation coefficients will be used to summarize and analyze the data. When categorical/binary outcomes are considered, chi-square test, Fisher's exact test, and logistic regression (incl. proportional odds and continuation ratio models) will be employed to examine, for example, the relationship between BMI (normal/overweight/obese) and groups (control/SS/NASH). When multiple variables are considered simultaneously main effects and interactions between factors will be tested via regression models.

For comparisons across groups, analysis of variance will first be performed, and if the null hypothesis is rejected, multiple range tests (e.g. Scheffe's, Least Significant Difference, and Newman-Keuls methods) will be conducted to identify significant differences between groups.

For all analyses, the Type I error will be set to 5%, i.e. alpha=0.05, for all calculations of confidence intervals and for performing the various hypothesis-testing procedures. Model diagnostic tools and cross-validation methods[275] will be considered for model assessment. Statistical analyses will be carried out using the statistical software packages SAS Version 9.2, and R Version 2.11.

(b) Specifically, to assess the link between intestinal microbiome and NAFLD, the following analyses will be performed:

Analysis of Variance and the Krusal-Wallis test, when applicable, will be used to compare F/B, and other IM-related measures, between groups (SS/NASH/Control). If the null hypothesis is rejected, pair wise comparisons will be employed to determine specific groups' differences, while using the Tukey-Kramer method for adjusting multiple comparisons. The relationship of an outcome variable, e.g. abundance of Firmicutes, with other variables such as age, gender, BMI and others, will be investigated using multiple regression methods. For metagenomics data, category-based clustering of the functions from each microbiome will be performed using principal component analysis (PCA) and hierarchical clustering.[276]

Bootstrap analyses will be used to identify metabolic pathways that were enriched or depleted in the microbiome-derived sequence bins.[277] (*More details are given under Data Mining*).

Characterization of the Intestinal Microbiome

The analysis of the microbiome in this project will produce complex data that must be integrated at various levels, for example microbial composition, disease and confounding factors such as BMI and diet. Furthermore, the data generated from microbiome data is vast and there are challenges with data analysis that can only be conducted using a Unix platform which we will use. Drs Krause & Stothard have extensive experience with working with large volumes of data. ***To develop the association between microbiome and the other variables, a biphasic approach will be followed; a data -mining phase and a hypothesis testing phase (see Figure)***

Multi-step approach for microbiome analysis

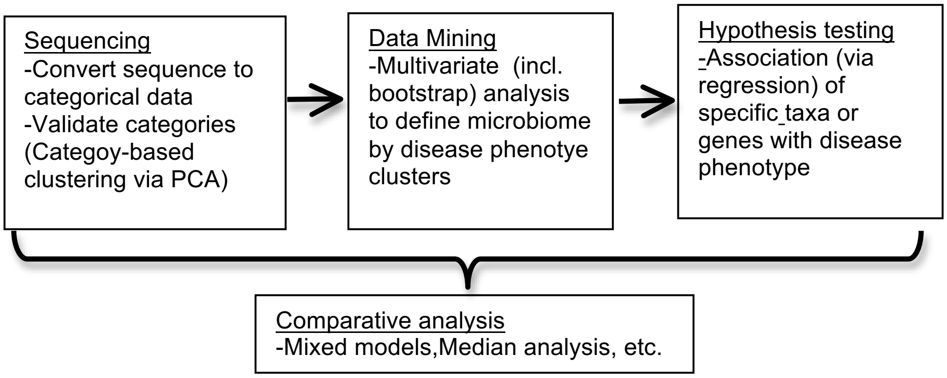

Data mining: This is a multivariate statistical approach used to associate data into groups. Clustering methods do not use predefined groups but other approaches use predefined groups; we will use and evaluate both. For example we will use association rule learning to explore the association between diet (high vs. low omega 3) and the microbiome. For instance the rule {NASH, omega 3 fatty acids} ==> {Bacteroidetes} indicates that if an individual has NASH and consumes a high omega 3 diet, then the likelihood of having high numbers of Bacteroidetes in the gut are good. To use this approach we will convert microbiome 16S rDNA phylogenetic data into categorical data. Categories will be defined as microbial taxons.

A key part of the data mining analysis is to identify groups of subjects that will be sequenced to obtain the metagenome. Cluster analysis of the microbiome will cluster subjects into groups that have the most similar microbiome. We will for example explore the relationships between diet and various families of glycosyl hydrolases (GH = carbohydrate degrading enzymes) and in this example the gene frequency data of GH families in the metagenome will be analyzed as above. For example, { NAFLD, omega 3 } ==> {Family IV GH} suggests that if one has NAFLD and you consume a high omega 3 diet then the likelihood that you have a high prevalence of genes in Family IV GH is high. This can then be expanded to the form {NAFLD, NASH} ==> {Bacteroidetes, Family IV GH } which indicates that Family IV GH systems from Bacteroidetes are the most significant.

Once data mining is complete, hypothesis testing proceeds and specific association are tested using robust statistical models. This is an important procedure because multivariate analyses often over-fit the data. We will use R, an open source programming language for statistics that is

highly scalable. For example, we will explore ANOVA type models to statistically test the association between Bacteroidetes (B), having NAFLD (N), NASH (SH) and Family IV GH (IV) genes. One approach is to use logistic regression analysis to define which variable should be in the model. The model B = NA+SH+IV may only account for 50% of the variation with a P-value of 0.08, and a reduced model without NASH may account for 83% of the variation and have a P-value of 0.001. This association also indicates that genes that encode Family IV GH from Bacteroidetes are statistically associated with NAFLD without NASH (i.e. only SS).

One of the statistical challenges is that microbiome data is not normally distributed and it is difficult to analyze. Because microbiome analysis is a fairly new area we will explore alternative analytical techniques. For instance we (Krause lab) have a full-time post-doctoral scientist working on developing Markov-Chain Monte Carlo (MCMC) methods for categorical microbiome data. MCMC are one way of solving the problem with defining a statistical distribution, the long tail in microbiome data, and low incidence data (lots of 1's and O's).

Specific bioinformatic analyses: Reads from metagenomic sequencing of pooled DNA (separate pools for each disease state and healthy individuals) will be filtered and trimmed, and the reads of each pool will be assembled into contigs using existing tools such as EULER,[230] to facilitate open reading frame (ORF) identification. ORFs will be predicted using MetaGene.[231] To speed up subsequent annotation, ORFs appearing to represent the same gene (based on stringent identity and overlap criteria) will be grouped, and the longest representative will be translated and annotated. The number of reads in each ORF group will be recorded for each sample, for use in the statistical comparisons described below. Annotation will involve assigning a functional class, pathway membership (i.e. which metabolic and signaling pathways include the protein), and source taxonomy (e.g. source species) whenever possible. Functional classification and pathway membership will be predicted using BLASTP[232] and the eggNOG database.[233] Source taxonomy will be predicted using BLASTN,[232] MEGAN, [234] and a subset of GenBank consisting of taxonomically assigned bacterial sequences, including all fully sequenced bacterial genomes. ORFs missing annotations at this stage will be subjected to a more computationally intensive analysis procedure involving more diverse databases (including, for example, all known phage proteins) and more sensitive comparison methods, such as PSI-BLAST[232] and HMMER.[235] The abundance of each functional class, pathway, and taxonomic group will then be compared between each disease state metagenome and the healthy metagenome, using the statistical methods implemented in STAMP[236] and Metastats.[237] We expect any taxonomic differences detected using this approach to be consistent with those found using 16S. Primers will be designed to target specific genes or gene groups identified by the 16S rDNA or metagenomic analysis as differing among individuals or pools, and quantitative real-time PCR will be used to validate the findings.

All metagenomics data and metadata will be collected and formatted according to the minimum information about a genome sequence (MIGS) specification recommended by the Genomic Standards Consortium.[238] All sequence analysis will be performed using command-line tools installed on a 64-processor Linux cluster. Although web-based metagenomics tools are available, these are not practical for large datasets or large numbers of samples, as transferring the massive data sets can be problematic, full control over methods and parameters is not available (for example, the comparison databases may be updated without warning), and analyses are not easily automated using scripts. The pipeline created through this work can be updated as new tools and methods are developed, and will be available for future work dealing with additional samples.

Interaction maps: In addition we are planning to construct interaction maps between hepatic gene expression and IM composition as well as between hepatic gene expression and metagenome.

Feasibility and Organizational Structure of the Research Group.
Feasibility(Tables 3,4, Appendix)
Enrolment: At least 130 patients with NAFLD (include simple steatosis and NASH) and 90 patients with NASH are diagnosed every year. Based on a previous study with UDCA, it is estimated that 50-75% of the patients screened for the study can be recruited: 50-75% of at least 130 patients per year over 2 years period = 130-195 potential patients. In the UDCA study, Dr. Heathcote enrolled asymptomatic patients with NASH in a 2-year randomized controlled trial with UDCA vs placebo involving 2 liver biopsies (baseline and 2 y). Twenty-eight patients with NASH were approached and screened for the study, 21 (75%) were enrolled and 15 (71%) completed the study with a second liver biopsy (drop-out 29%). Among the patients with NASH, 50% were female, 67% had a BMI > 30 kg/m^2, 25% had diabetes mellitus and 50% hyperlipidemia. Therefore, we anticipate meeting our target within a 2-year Grant. In addition, since there is no specific effective therapy for this disease, gastroenterologists in the community are very willing to refer patients with suspected NAFLD to hepatology clinics, especially when a study is taking place.
Laboratory: Analyses used in this grant are presented in Table 3 , Fig 1,2, or in papers in the Appendix.
These analyses are presented to support the feasibility of the project. 1. Blood glucose, insulin and c-peptide were measured in a study investigating the chromium status of HIV patients in relation to abnormal lipid and glucose metabolism (funded by CANFAR). Insulin Resistance Index (IRI) was then calculated by adding the Log of serum fasting insulin to the log of serum fasting glucose (Table 3A). 2. As part of another HIV study in collaboration with Dr. Alice Tang, Tufts University, Boston, we also previously performed the analysis of plasma antioxidant power (Table 3B). 3. In a pilot study, pathology and immunochemistry (TGF-β, α-SMA) were performed on liver biopsies of a small group of patients with simple steatosis (no NASH) and NASH (Table 3C, Fig 1,2) using a validated scoring system (199). Overall, α-SMA scores were higher in the NASH group. For TGF-β, most patients with steatosis (no NASH) and NASH scored 1+ with one patient with the highest intensity score having NASH. 4. On these liver biopsies, we also measured both TNF-alpha and lipid peroxides (Table 3D). The size of the biopsy specimen allowed us to do both measurements. In this small group, those with NASH had a significantly (P=0.006) higher lipid peroxides and a trend (P= 0.080) toward a higher TNF-alpha compared with simple steatosis (no NASH). Other measures of oxidative stress are shown in appended publications (Table 3E, Appendix). These analyses in addition to nutritional assessment (dietary intake analyses, plasma micronutrients, body composition) have been performed in many of our studies (Table 3E, Appendix).
Organizational Structure: All the co-investigators (see feasibility and CV) have the staff and the facilities to conduct this study. All the liver biopsies will be reviewed by one pathologist (Dr. Guindi). Except for standard laboratory analysis, all other specimens will be stored at –70 C in Dr. Allard's laboratory at The Toronto General Hospital. IM Analyses will be performed in the laboratories of Dr. Elena Comelli (University of Toronto) and Dr. Denis Krause (University of Manitoba, Winnipeg). Bioinformatic analyses will be performed by Dr. Krause and Dr. Paul Stothard (University of Alberta, Edmonton).

RELEVANCE / FUTURE RESEARCH

NAFLD is the most common cause for abnormal liver tests in North American adults. Simple steatosis is a benign condition but NASH may progress to cirrhosis. Obesity, diabetes and hyperinsulinemia have been associated with NAFLD. However, many individuals lack the typical risk factors and no one knows why some patients have an indolent course while others developed complications. The results of this study will help elucidate the role of lipid peroxidation in the pathogenesis of NASH and determine if there is an association between lipid peroxidation and lower antioxidant status, increased liver cytokines (TNF-α, TGF-β), insulin resistance and higher unsaturated fatty acid status. It also helps us assess whether tere is a difference in fatty acid composition of the liver and in hepatic gene expression between NASH, SS and healthy control subjects. In addition, patients with non-alcoholic fatty liver disease (SS+NASH) with low hepatic n03 PUFA will be compared to those with high n-3 PUFA content in the liver to determine if there is a difference in expression of the genes involved in lipid metabolism and oxidative stress. Also, more dietary studies are required since some of the factors influencing n-3 PUFA content in the liver are dietary imbalance such as low n-3 PUFA, high trans fat or low antioxidants intakes which can also lead to high hepatic oxidative stress. The results from this study may lead to further studies on the effect of specific dietary intervention such as increased n-3 PUFA intake or supplementation. The microbiota composition will produce pilot data for further assessment of the role of microbiota in NAFLD and possible interventions with agents like antibiotics or probiotics.

REFERENCES

1. Sanson-Fisher R. The measurement of alcohol consumption and alcohol related problems. Aust Drug Alcohol Rev 1986, 5:309-313.
2. Poucell S, Ireton J, Valencia-Mayoral P et al. Amiodarone-associated phospholipidosis and fibrosis of the liver. Light, immunohistochemical and electron microscopic studies. Gastroenterol 984, 86:926-36.
3. Pessayre D, Bichara M, Degott C et al. Perhexilene maleate-induced cirrhosis. Gastroenterol 1979, 76:170-7.
4. Farrell GC. Steatohepatitis. In: Farrell GC ed. Drug-induced liver disease. Edinburgh: Churchill Livingstone: 1995, p. 431-8.
5. George DK, Goldwurm S, MacDonald GA et al. Increased hepatic iron concentration in nonalcoholic steatohepatitis is associated withincreased fibrosis. Gastroenterol 1998, 114:311-318.
6. Van Ness MM, Diehl AM. Is liver biopsy useful in the evaluation of patients with chronically elevated liver enzymes? Ann Intern Med 1989; 111:473-8.
7. Diehl AM, Goodman Z, Ishak KG. Alcohol-like liver disease in nonalcoholics. A clinical and histopathological comparison with alcohol-induced liver injury. Gastroenterology 1989, 95:1056-1060.1989.
8. Ludwig J, Viggiano RT, McGill DB. Nonalcoholic steatohepatitis: Mayo Clinic experiences with a hitherto unnamed disease. Mayo Clin Proc 1980, 55:342-348.
9. Powell EE, Cooksley GE, Hanson R et al. The natural history of nonalcoholic steatohepatitis: a follow-up study of 42 patients follow for up to 21 years. Hepatology 1990, 11:74-80.
10. Lee RG. Nonalcoholic steatohepatitis: a study of 49 patients. Hum Pathol 1989, 20: 594-599.
11. Leevy CM. Fatty liver: a study of 270 patients with biopsy proven fatty liver and a review of the literature. Medicine (Baltimore) 1962, 4:249-258.
12. Bacon B, Faravash MJ, Janney CG, et al. Nonalcoholic steatohepatitis: an expanded clinical entity. Gastroenterology 1994, 107:1103-1106.
13. Byron D, Minuk G. Clinical hepatology: profile of an urban, hospital-based practice.

Hepatology 1996;24:813-15.
14. Itoh S, Igarashi M, Tsukada T, et al. Nonalcoholic fatty liver with alcoholic hyaline ate long-term glucocorticoid therapy. Acta Hepatogastroenterol 1977, 24:415-19.
15. Lee RG. Nonalcoholic steatohepatitis: tightening the morphological screws of a hepatic rambler. Hepatoogy 1995, 21:1742-44.
16. Falchuk KR, Fiske SC, Haggit RC et al. Pericentral hepataic fibrosis and intracellular hyaline in diabetes mellitus. Gastroenterology 1980, 78:535-540.
17. Silverman JF, O'Brien KF, Long S, et al. Liver pathology in morbidly obese patients with and without diabetes. Am J Gastroenterol 1990, 85:1345-1355.
18. Angulo P. Nonalcoholic fatty liver disease. N Engl J Med 2002, 346: 1221-1231.
19. Sheth SG, Gordon FD, Chopar S. Nonalcoholic steatohepatitis. Ann Intern Med 1997, 126:137-145.
20. Teli M, Oliver FW, Burt AD et al. A natural history of nonalcoholic fatty liver: a follow-up study. Hepatology 1995, 22:1714-1717.
21. Berkowitz D. Metabolic changes associated with obesity before and after weight reduction. J Am Med Assoc 1964, 187:103-110.
22. Palmer M, Schaffner F. Effect of weight reduction on hepatic abnormalities in overweight patients. Gastroenterol 1990, 99:1408-1411.
23. Kamimura S, Tsukamoto H. Cytokine gene expression by Kuppfer cells in experimental alcoholic liver disease. Hepatology 1995, 22:1304-1309.
24. Luyckx FH, desaive C, Thiry A, et al. Liver abnormalities in severly obese subjects: effect of drastic weight loss after gastroplasty. Int J Obes Relat Metab Disord 1998; 22:222-6.
25. Limuro Y, Gallucci RM, Luster MI et al. Antibodies to tumor necrosis factor alfa attenuates hepatic ncrosis and inflammation caused by chronic exposure to ethanol in the rat. Hepatology 1997, 26:1530-37.
26. Uysal KT, Wiesbrock SM, Marino MW et al. Protection from obesity-induced insulin-resistance in mice lacking TNF alpha function. Nature 1997, 389:610-614.
27. Marchesini G, Brizi M, Morselli-Labate AM et al. Association of non-alcoholic fatty liver disease with insulin resistance. Am J Med 1999, 107:450-455.
28. Batman PA, Scheuer PJ. Diabetic hepatitis preceding the onset of glucose intolerance. Histopathol 1985, 9:237-243
29. Shimomura I, Hammer RE, Ikemoto S et al. Leptin reverses insulin resistance and diabetes mellitus in mice with congenital lipodystrophy. Nature 1999, 401: 73-76.
30. Yang SQ, Lin HZ, Lane MD et al. Obesity increases sensitivity to endotoxin liver injury: implications for the pathogenesis of steatohepatitis. Proc Ntl Acad Sci USA 1997, 94:2557-62.
31. Loffreda S, Yang SQ, Lin HZ et al. Leptin regulates proinflammatory immune responses. FASEB J 1998, 12:57-65.
32. Capron JP, Delamarre M, Dupas JL et al. Fasting in obesity: another cause of liver injury with alcoholic hyaline? Dig Dis Sci 1982, 54:374-377.
33. Lavine JE. Vitamin E treatment of nonalcoholic steatohepatitis in children: a pilot study. J Pediatr 2000, 136:734-8.
34. Hasegawa T, Yoneda M, Nakamura K et al. Plasma transforming growth factor-beta1 level and efficacy of alpha-tocopherol in aptients with non-alcoholic steatohepatitis: a pilot study. Aliment Pharmacol Ther 2001;15:1667-1672.
35. Diehl AM. Nonalcoholic Steatohepatitis. Seminars in Liver Disease 1999, 19:221-229.
36. Reid AE. Nonalcoholic steatohepatitis. Gastroenterology 2001;121:710-23.
37. James OFW, Day CP. Non-alcoholic steatohepatitis (NASH): a disease of emerging identity and importance. J Hepatol 1998, 29:495-501.
38. Matteoni CA, Younossi ZM, Gramlich T, Boparai N, Liu YC, McCullough AJ. Nonalcoholic fatty liver disease: a spectrum of clinical and pathological severity. Gastroenterology 1999; 116:1413-9.
39. Angulo P, Keach JC, Batts KP, Lindor KD. Independent predictors of liver fibrosis in patients with nonalcoholic steatohepatitis. Hepatology 1999; 30:1356-62.

40. Rtaziu V, Giral P, Charlotte F, et al. Liver fibrosis in overweight patients. Gastroenterology 2000; 118:1117-23.
41. Manton ND, Lipsett J, Moore DJ, Davidson GP, Bourne AJ, Couper RTL. Non-alcoholic steatohepatitis in children and adolescents. Med J Aust 2000; 173:476-9.
42. Braillon A, Capron JP, Herve MA, et al. Liver in obesity. Gut 1985, 26:133-139.
43. Ruderman N, Chisholm D, Pi-Sunyer X, Schneider S. The metabolically obese, normal weight individual revisited. Diabetes 1998; 47:699-713.
44. Bellentani S, Saccoccio G, Masutti F, et al. Prevalence of and risk factors for hepatic steatosis in northern Italy. Ann Intern Med 2000; 132:112-7.
45. Wanless IR, Lentz JS. Fatty liver hepatitis (steatohepatitis) and obesity: an autopsy study with analysis of risk factors. Hepatology 1990, 12:1106-1110.
46. Assy N, Kaita K, Mymin D, Levy C, Rosser B, Minuk G. Fatty infilteration of liver in hyperlipidemic patients. Dig Dis sci 2000; 45:1929-34.
47. Moran JR, Ghishan FK, Halter SA et al. Steatohepatitis in obese children: a cause of chronic liver dysfunction. Am J Gastroenterol 1983, 78:374-7.
48. Baldridge AD, Perez-Atayde AR, Graeme-Cook F et al. Idiopathic steatohepatitis in childhood: a multicentre retrospective study. J Pediatr 1995, 127:700-4.
49. Rashid M, Roberts EA. Non-alcoholic steatohepatitis in children. Journal of Pediatric Gastroenterology and Nutrition 2000;30:48-53.
50. Roberts EA. Steatohepatitis in children. Best Practice & Research Clinical Gastroenterology. 2002;16(5):000 (In Press).
51. Nomura H, Kashiwagi S, Hayashi J, Kajiyama W, Tani S, Goto M. Prevalence of fatty liver in a general population of Okiawa, Japan. Jpn J Med 1988; 27:142-149.
52. Tominaga K, Kurata JH, Chen YK, et al. Prevalence of fatty liver in Japanese children and relationship to obesity: an epidemiological ultrasonographic survey. Dig Dis Sci 1995; 40:2002-9.
53. Franzese A, vajro P, Argenziano A, et al. Liver involvement in obese children: ultrasonography and liver enzyme levels at diagnosis and during follow-up in an Italian population. Dig Dis Sci 1997;42: 1428-32.
54. Daniel S, Ben-Menachem T, vasudevan G, Ma CK, Blumenkehl M. prospective evaluation of unexplained chronic liver transaminase abnormalities in asymptomatic and symptomatic patients. Am J Gatroenterol 1999; 94:3010-4.
55. Clark JM, Brancati FL, Diehl AME. Nonalcoholic fatty liver disease: The most common cause of abnormal liver enzymes in the U.S. population. Gastroenterology 2001; 120: supple: A-65. Abstract.
56. Flegal KM, Carroll MD, Kuczmarski RJ, Johnson CL. Overweight and obesity in the United States: prevalence and trends, 1960-1994. Int J Obes Relat Metab Disord 1998; 22:39-47.
57. Profiles of general demographic characteristics: 2000 Census of population and housing. Washington, D.C.: Bureau of the Census, May 2001.
58. Silverman JF; Pories WJ, Caro JE. Liver pathology in diabetes mellitus and morbid obesity: clinical, pathological and biochemical considerations. Pathol Annu 1989; 24:275-302.
59. Alter MJ, Kruszon-Moran D, Nainan OV, et al. The prevalence of hepatitis C virus infection in the United States, 1988 through 1994. N Engl J Med 1999; 341:556-62.
60. National Population Health Survey 1994-1995. Ottawa: Statistics Canada:1995
61. Adler M, Schaffner F. Fatty liver hepatitis and cirrhosis in obese patients. Am J Med 1979, 67:811-9.
62. Itoh S, Yougel T, Kawagoe K. Comparison between nonalcoholic steatohepatitis and alcoholic hepatitis. Am J Gastroenterol 1987, 82:650-4.
63. Diehl AM, Goodman Z, Ishak KG. Alcohol-like liver disease in nonalcoholics. A clinical and histopathological comparison with alcohol-induced liver injury. Gastroenterol 1988, 95: 1056-1060.
64. Laurin J, Lindor KD, Crippin JS et al. Ursodeoxycholic acid or clofibrate in the treatment of non-alcoholic induced steatohepatitis: a pilot study. Hepatology 1996, 23:1464-7.
65. Pinto HC, Baptista A, Cailo ME et al. Nonalcoholic steatohepatitis. Clinicopathological comparison with alcoholic hepatitis in ambulatory and hospitalized patients. Dig Dis Sci 1996, 41:172-9.

66. Hilden M, Juhl E, Thomen AC et al. Fatty liver persisting for up to 33 years. Acta Med Scand 1973, 194:485-9.
67. Propst A, Propst T, Judmaier G et al. Prognosis in nonalcoholic steatohepatitis [letter]. Gastroenterol 1995, 108:1607.
68. Shelden GF, Petersen SR, Sanders R. Hepatic dysfunction during hyperalimentation. Arch Surg 1978, 113:504-508.
69. Peden VH, Witzelben CI, Skelton MA. Total parenteral nutrition. J Pediatr 1971, 78:180 (letter).
70. Nussbaum MS, Fisher JE. Pathogenesis of hepatic steatosis during total parenteral nutrition. Surg Annu 1991, 2:1-18.
71. Freund HR, Muggia-Sullam M, LaFrance R et al. A possible beneficial effect of metronidazole in reducing TPN-associated liver function derangements. J Surg 1985, 38:356-60.
72. Letter on P, Fromenty B, Terris B et al. Acute and chronic hepatitic steatosis lead to in vivo lipid peroxidaiton in mice. J Hepatol 1996, 24:200-8.
73. Jaeschke H, Wang Y, Essani NA. Reactive oxygen species activate the transcription factor NF-kB in the liver by induction of lipid peroxidation [abstract]. Hepatology 1996, 24:238A.
74. Day CP, James OFW. Steatohepatitis: a tale of two "hits"? Gastroenterol 1998, 114:842-45.
75. James O, Day C. Non-alcoholic steatohepatitis: another disease of affluence. The Lancet 1999, 353:1634-1636.
76.Day C, James O. Hepatic steatosis: innocent bystander or guilty party? Hepatology 1998;27:1463-1466.
77.Reynet C, Kahn CR. Rad: a member of the Ras family over expressed in muscle of type II diabetic humans. Science 1993; 262:1441-4.
78.Maddux BA, Sbraccia P, Kumakura S, et al. Membrane glycoprotein PC-1 and insulin resistance in non-insulin dependent diabetes mellitus. Nature 1995; 373:448-51.
79.Cohen B, Novick D, Rubinstein M. Modulation of insulin activities by leptin. Science 1996; 274:1185-8.
80.Boden G. role of fatty acids in the pathogenesis of insulin resistance and NIDDM. Diabetes 1997; 46:3-10. [Erratum, Diabetes 1997; 46:536.]
81.Hotamisligi GS, Peraldi SP, Budavari A, Ellis R, White MF, Spiegelman BM. IRS-1-mediated inhibition of insulin receptor tyrosin kinase activity in TNF-alpha- and obesity-induced insulin resistance. Science 1996; 271:665-8.
81a. Bass NM. Three for the price of one knockout-a mouse model of congenital peroxisomal disorder, steatohepetitis and hepatocarcinoma. Hepatology 1999; 29:606-07.
82.Fong D, Nehra V, Lindor K et al. Metabolic and nutritional considerations in nonalcoholic fatty liver. Hepatology 2000;32:3-10.
83. Van Steenbergen W, Lanckmans S. Liver disturbances in obesity and diabetes mellitus. Int J Obes Relat Metab Disord 1995;19(suppl 3):S27-S36.
84. Fisher R. Hepatobiliary abnormalities associated with total parenteral nutrition. Gastroenterol Clin North Am 1989;18:645-666.
85. McLaren D, Bitar J, Nassar V. Protein-calorie malnutrition and the liver. Prog Liver Dis 1972;4:527- 536.
86. McGarry J, Foster D. Regulation of hepatic fatty acid oxidation and ketone body production. Annu Rev Biochem 1980;49:395-420.
87. Osmundsen H, Bremer J, Pedersen J. Metabolic aspects of peroxisomal b-oxidation. Biochem Biophys Acta 1991;1085:141-158.
88. Weltman MD, Farrell GC, Hall P et al. Hepatic Cytochrome P450 2E1 is increased in patients with nonalcoholic steatohepatitis. Hepatology 1998, 27:128-133.
89. Leclercq IA, Farrell GC, Field J et al. CYP2E1 and CYP4A as microsomal catalysts of lipid peroxides in murine nonalcoholic steatohepatitis. J Clin Invest 2000, 105: 1067-1075.
90. Sanyal AJ, Campbell-Sargent C, Mirshahi F et al. Nonalcoholic steatohepatitis: association of insulin resistance and mitochondrial abnormalities. Gastroenterol 2001, 120:1183-1192.
91. Baeulrle PA, Henkle T. Function and activation of NF_kB in the immune system. Annu Rev Immunol 1994, 12:141-179.

92. Libermann TA, Baltimor D. Activation of interleukin-6 gene expression through the NF-kB transcription factor. Mol Cell Biol 1990; 10: 2327-2334.
93. Shakhov AN, Collart WA, Vassalli P, et. Al. KB-type enhancers are involved in lipopolysaccharide- mediated transcriptional activation of the tumor necrosis factor alpha gene in primary macrophages. J Exp Med 1990; 171: 35-47.
94. Hotamisligil GS, Spiegerman BM. Adipose expression of tumor necrosis factorα: direct role in obesity-linked insulin resistance. Science 1993, 259:87-91.
95. Qi C, Pekala PH. Tumor necrosis factor-alpha-induced insulin resistance in adipocytes. Exp Biol & Med 2000, 223:128-35.
96. Hotamisligil GS, Arner P, Caro JF et al. Increased adipose expression of tumor necrosis Factor α in human obesity and insulin resistance. J Clin Invest 1995, 95:2409-2415.
97.Kral J, Schaffner F, Pierson R et al. Body fat topography as an independent predictor of fatty liver. Metabolism 1993;42:548-551.
98. Matsuzawa Y, Shimomura I, Nakamura T et al. Pathophysiology and pathogenesis of visceral fat obesity. Obes Res 1995;3(suppl 2):187s-194S.
99. Jensen MD. Diet effects on fatty acid metabolism in lean and obese humans. Am J Clin Nutr 1998, 67(suppl):531s-534s
100. Kissebah A, Vydelingum N, Murray R et al. Relation of body fat distribution to metabolic complications of obesity. J Clin Endocrinol Metab 1982, 54:254-60.
101. Jensen MD, Haymond MW, Rizza RA et al. Influence of body fat distribution on free fatty acid metabolism in obesity. J Clin Invest 1989, 83:1168-73.
102. Arner P. Not all fat is alike. Lancet 1998, 351:13.
103. Dobrian AD, Davies MJ, Schriver SD et al. Oxidative stress in a rat model of obesity-induced hypertension. Hypertension, 2001, 37:554-60.
104. Beltowski J, Wojcicka G, Gorny D et al. The effect of dietary-induced obesity on lipid peroxidation, antioxidant enzymes and total plasma antioxidant capacity. J Physiol and Pharmacol, 2000, 51:883-96.
105. Laight DW, Desai KM, Gopaul NK et al. F2-isoprostane evidence of oxidant stress in the insulin resistant, obese Zucker rat: effects of vitamin E. Eur J Pharmacol 1999, 377:89-92.
106. Dandona P, Mohanty P, Ghanim H et al. The suppressive effect of dietary restriction and weight loss in the obese on the generation reactive oxygen species by leukocytes, lipid peroxidation and protein carbonylation. J Clin Endocrinol & Metab 2001, 86:355-62.
107. Heini AF, Lara-Castro C, Kirk KA et al. Association of leptin and hunger-satiety ratings in obese women. Int J Obes Relat Metabol Disord 1998, 22: 1084-1087.
108. Martin LJ, Jones PJ, Considine RV et al. Serum leptin levels and energy expenditure in normal weight women. Can J Physiol Pharmacol 1998, 76:237-241.
109. Wadden TA, Considine RV, Foster GD, et al. Short- and long-term changes in serum leptin dieting obese women: effects of caloric restriction and weight loss. J Clin Endocrinol Metab 1998, 83:214-218.
110. Considine RV, Sinha MK, Heiman ML et al. Serum immunoreactive-leptin concentrations in normal-weight and obese humans. N Engl J Med 1996, 334:292-295.
111. Boulimie A, Marumo T, Lafontan M et al. Leptin induces oxidative stress in human endothelial cells. FASEB J 1999, 13:1231-8.
112. Strauss RS. Comparison of serum concentrations of alpha-tocopherol and beta-carotene in a cross- sectional sample of obese and non-obese children (NHANES III). J Pediatr 1999, 134:160-5.
113.Barak AJ, Beckenhauer HC, Badakhsh S et al. The effect of betaine in reversing alcoholic steatosis. Alcohol Clin Exp Res 1997, 21:1100-2.
114. Ohrvall M, Tengblad S, Vessby B. Lower tocopherol serum levels in subjects with abdominal adiposity. J Intern Med 1993, 234:53060.
115. Viroonudomphol D, Pongpaew P, Tungtrongchitr R et al. Erythrocyte antioxidant enzymes and blood pressure in relation to overweight and obese Thai in Bangkok. South Asian J Trop Med & Public Health 2000, 31:325-34.
116. Moor de Burgos A, Wartanowics M, Ziemlanski S. Blood vitamin and lipid levels in overweight and obese women. Eur J Clin Nutr1992;46:803-8.

117. Kuno T, Hozumi M, Morinobu TM et al. Antioxidant vitamin levels in plasma and low density lipoprotein of obese girls. Free Radic Res 1998;28:81-6.
118. Decsi T, Molnar D, Koletzo B. Reduced plasma concentrations of alpha-tocopherol and beta- carotene in obese bous. J Pediatr 1997;130:653-5.
119. Tsukamoto H, Horne W, Kamimura S et al. Experimental liver cirrhosis induced by alcohol and iron. J Clin Invest 1995, 96: 620-30.
120. Weltman MD, Farrell GC, Liddle C. Increased hepatocyte CYP2E1 expression in a rat nutitional model of hepatic steatosis with inflammation. Gastroenterol 1996, 111: 1645-53.
121. Woodcroft KJ, Novak RF. Insulin effects on CYP2E1, 2B, 3A and 4A expression in primary cultured rat hepatocytes. Chem Biol Interact 1997, 107:75-91.
122. Lieber CS. Cytochrome P-4502E1: its physiological and pathological role. Physiol Res 1997, 77:517-544.
123. Ekstrom G, Ingelman-Sundberg M. Rat liver microsomal NADPH-supported oxidase activity and lipid peroxidation dependent on ethanol-inducible cytochrome P-450 (P-450IIE1). Biochem Pharmacol 1989, 38: 1313-1319.
124. Berson A, de Beco C, Letteron P et al. Steatohepatitis-inducing drugs cause mitochondrial dysfunction and lipid peroxidaiton in rat hepatocytes. Gatsroenterology 1998;114:764-774.
125. Nelson DR. Metazoan cytochrome P450 evolution. Comp Biochem Physiol C Pharmacol Toxicol Endocrinol 1998, 121:15-22.
126. Lee KS, Buck M, Houglum K et al. Activation of hepatic stellate cells by TGF alpha and collagen type I is mediated by oxidative stress through c-myb expression. J Clin Invest 1995;96:2461-8.
127. Parola M, Pinzani M, Casini A et al. Stimulation of lipid peroxidation or 4hydroxynonenal treatment increases procollagen alpha 1 (I) gene expression in hman liver fat-storing cells. Biochem Biophys Res Commun 1993;194:1044-50.
128. Bedossa P, Houglum K, Trautwein C et al. Stimulation of collagen alpha 1 (I) gene expression is associated with lipid peroxidation in hepatocellular injury: a link to tissue fibrosis? Hepatology 1994;19:1262-71.
129 Paradis V, Mathurin P, Kollinger M et al. In situ detection of lipid peroxidation in chronic hepatitis C: correlation with pathological features. J Clin Pathol. 1997;50:401-6.
130. Farinati F, Cardin R, De Maria N et al. Iron storage, lipid peroxidation and glutathione turnover in chronic anti-HCV positive hepatitis. J Hepatol 1995;22:449-56.
131. Houglum K, Ramm GA, Crawford DH et al. Excess iron induces hepatic oxidative stress and transforming growth factor beta-1 in genetic hemochromatosis. Hepatology 1997;26:605-10. 132. 132. Kamimura S, Gaal K, Britton RS et al. Increased 4-hydroxynonenal levels in experimental alcoholic liver disease: association of lipid peroxidation with liver fibrogenesis. Hepatology 1992;16:448-53.
133. Niemela O, Parkkila S, Yla Herttuala S et al. Covalent protein adducts in the liver as a result of ethanol metabolism and lipid peroxidation. Lab Invest 1994;70:537-46.
134. Yoshimura T, Matsushima K, Tanaka S, et al. Purification of a human monocyte-derived neutrophil chemotactic factor that has peptide sequence similarity to other host defense cytokines. Proc Natl Acad Sci U.S.A 1987; 84:9233-7.
135. Hamond ME, Lapointe GR, Feicht PH et al. IL-8 induces neutrophil chemotaxis predominantly via type 1 IL-8 receptors. J Immunol 1995, 155:1428-1434.
136. Mizuhara H, O'Neill E, Seki N et al. T cell activation-associated hepatic injury: mediation by tumor necrosis factors and protection by interleukin-6. J Exp Med 1994, 179:1529-37.
137. Orange JS, Salazar-Mather TP, Opal SM et al. Mechanisms for virus-inducde disease: tumor necrosis factor-mediated pathology independent of natural killer and T cells during murine cytomegalovirus infection. J Virol 1997, 71:9248-58.
138. Trauner M, Meier PJ, Boyer JL. Molecular pathogenesis of cholestasis. N Engl J Med 1998, 339:1217-27.
139. Friedman SL. Molecular regulation of hepatic fibrosis, an integrated cellular response to tissue injury. J Biol Chem 2000, 275:2247-50.
140. Higuchi M, Aggarwal BB, Yeh ETH. Activation of CPP32-like protease in tumor necrosis

factor- induced apoptosis is dependent on mitochondrial funtion. J Clin Invest 1997; 99:1751-8.
141. Inayat-Hussain SH, Couet C, Cohen GM, Cain K. Processing/activation of CPP32-like proteases is involved in transforming growth factor B1-induced apoptosis in rat hepatocytes. Hepatology 1997; 25:1516-26.
142. Leonarduzzi G, scavazza A, Biasi F, et al. The lipid peroxidation end product 4-hydroxy-2,3-nonenal up-regulates transforming growth factor b1 expression in the macrophage lineage: a link between oxidative injury and fibrosclerosis. FASEB J 1997; 11:851-7.
143. Hrudzkewycz AM. Evidence for mitochondrial DNA damage by lipid peroxidation. Biochem Piophys Res Commun 1988, 153:191-197.
144.Chen J, Schenker S, Frosto TA et al. Inhibition of cytochrome c oxidase activity by 4-hydroxy-nonenal (HNE). Role of HNE adduct formation with the enzyme catalytic site. Biochem Biophys Acta 1998, 1380:336-344.
145. Yang SQ, Zhu H, Li Y et al. Mitochondrial adaptation to obesity-related oxidant stress. Arch Biochem Biophys 2000, 378:259-268.
146. Pessayre D, Feldman G, Haousi D et al. Hepatocyte apoptosis triggered by natural substances (cytokines, other endogenous substances and foreign toxins). In: Cameron RG, Feuer G, eds. Apoptosis and its modulation by drugs. Heidelberg: Springer Verlag, Handbook Exp Pharmacol 2000, 142:59-108.
147. Schulze-Osthoff K, Bakker AC, Vanhaesebroeck B et al. Cytotoxic activity of tumor ncrosis factor is mediated by early damage of mitochondrial functions: evidence for the involvement of mitochondrial radical generation. J Biol Chem 1992, 267:5317-23.
148. Higuchi M, aggarwal BB, Yeh ET. Activation of CPP32-like protease in tumor necrosis factor-induced apoptosis is dependent on mitochondrial function. J Clin Invest 1997, 99: 1751-8.
149. Hug H, strand S, grambihler A, et al. Reactive oxygen intermediates are involved in the induction of CD95 ligand mRNA expression by cytostatic drugs in hepatoma cells. J Biol Chem 1997; 272:28191-3.
150. Capel ID, Dorrell HM. Abnormal antioxidant defense in some tissues of congenitally obese mice. Biochem J 1984, 219: 41-49.
151. Watson AM, Poloyac SM, Howard G et al. Effect of leptin on cytochrome p-450, conjugation and antioxidant enzymes in the ob/ob mouse. Drug Metab Dispos 1999, 27: 695-700.
152. Sastre J, Pallardo FV, Llopis J et al. Glutathione depletion by hyperphagia-induced obesity. Life Sci 1989, 45:183-187.
153. Douillet C, Bost M, Accominotti M et al. Effect of selenium and vitamin E supplementation on lipid abnormalities in plasma, aorta and adipose tissue of Zucker rats. Biol Trace Element Res 1998, 65:221-36.
154. Soltys K, Dikdan G, Koneru B. Oxidative stress in fatty livers of obese Zucker rats: rapid amelioration and improved tolerance to warm ischemia with tocopherol. Hepatology, 2001, 34(1): 13-8.
155. Garcia-Ruiz C, Morales A, Colell A et al. Feeding S-adenosyl-L-methionine attenuates both ethanol-induced depletion of mitochondrial glutathione and mitochondrial dysfunction in periportal and perivenous rat hepatocytes. Hepatology 1995, 21:207-14.
156. Nanji AA, Yang EK, Fogt F et al. Medium chain triglycerides and vitamin E reduce the severity of established experimental alcoholic disease. J Pharmacol Exp Ther 1996, 277:1694-700.
157. Moor de Burgos A, Wartanowics M, Ziemlanski S. Blood vitamin and lipid levels in overweight and obese women. Eur J Clin Nutr 1992, 46:803-8.
158. Meyer M, Schreck R, Baeuerle PA. H2O2 and antioxidants have opposite effects on activation of NF-kB and AP-1 in intact cells: AP-1 as secondary-responsive factor. EMBO J 1993; 12:2005- 2015.
159. Suzuki YJ, Pecker L. Inhibition of NF-kB activation by vitamin E derivatives. Biochem Biophys Res Commun 1993; 193: 277-283.
160. Suzuki YJ and Pecker L. Inhibition of NF-kB DNA binding activity by alpha-tocopheryl

succinate. Biochem Mol Biol Int 1993; 31:693-700.
161. Meyer M, Pahl HL, Baeuerle PA. Regulation of the transcription factors NF-kappaB and AP- 1 redox changes. Chem Biol Interact 1994, 91:91-100.
162. Islam KN, Devaraj S, Jialal I. Alpha-tocopherol enrichment of monocytes decreases agonist- induced adhesion to human endothelial cells. Circulation 1998, 98:2255-61.
163. Freedman JE, Farhat JH, Loscalzo J et al. Alpha-tocopherol inhibits aggregation of human platelets by a protein kinase C-dependent mechanism. Circulation 1996, 94:2434-40.
164. Devaraj S, Li D, Jialal I. The effects of alpha-tocopherol supplementation on monocyte function. Decreased lipid oxidation, interleukin 1 beta secretion, and monocyte adhesion to endothelium. J Clin Invest 1996, 98:756-63.
165. Kanno T, Utsumi T, Kobuchi H et al. Inhibition of stimulus-specific neutrophil superoxide generation by alpha-tocopherol. Free Radic Res 1995, 22:431-40.
166. Boscoboinik D, Szewczyk A, Hensey C et al. Inhibition of cell proliferation by alpha-tocopherol. Role of protein kinase C. J Biol Chem 1991, 266:6188-94.
167. Fox ES, Brower JS, Bellezzo JM et al. N-acetylcysteine and alpha-tocopherol reverse the inflammatory response in activated rat Kuppfer cells. J Immunol 1997, 158:5418-23.
168. Akeson AL, Woods CW, Mosher LB et al. Inhibition of IL-1 beta expression in THP-1 cells by probucol and tocpherol. Atherosclerosis 1991, 86:261-70.
169. Barve S, Joshi-Barve, Talwaker R, et.al. Mesalamine (5-ASA) and the antioxidant, vitamin E, inhibit interleukin-1 (IL-1) mediated activation of nuclear factor-kB (NF-kB) in ceco-2 cells. Gastroenterology 1995; 108: A777.
170. Droge W, Schulze-Osthoff K, Mihm S, et. Al. Functions of glutathione and glutathion disulphide in immunology and immunopathology. FASEB J 1994; 8: 1131-1138.
171. Packer L. Vitamin E is nature's master antioxidant. Sci Am Sci Med 1994, 1:54-63.
172. Mol MJ, de Rijke YB, Demacker PN et al. Plasma levels of lipid and cholesterol oxidation products and cytokines in diabetes mellitus and cigarette smoking: effects of vitamin E treatment. Atherosclerosis 1997, 129:169-76.
173. Neuschwander-Tetri et al. Rosiglitazone improves insulin sensitivity, ALT and hepatic steatosis in patients with nonalcoholic steatohepatitis. Abstract presented DDW April 2002, San Francisco.
174. Marchesini G, Brizi M, Bianchi G et al. Metformin in non-alcoholic steatohepatitis. Lancet 2001;358(9285):893-894.
175. Caldwell SH, Hespenheide EE, Redick JA et al. A pilot study of a Thiazolidinedione, Troglitazone, in non-alcoholic steatohepatitis. Am J Gastro 2001;96:519-525.
176. Gitlin N, Julie NL, Spurr CL et al. Two cases of severe clinical and histologic hepatotoxicity associated with troglitazone. Ann Intern Med 1998;129:36-8.
177. Neuschwander-Tetri BA, Isley WL, Oki JC, et al. Troglitazone-induced hepatic failure leading to liver transplantation. Ann Intern Med 1998;129:38-41.
178. Laurin J, Lindor KD, Crippin JS et al. Ursodeoxycholic acid or clofibrate in the treatment of non- alcoholic induced steatohepatitis: a pilot study. Hepatology 1996;23:1464-1467.
179. Abdelmalek MF, Angulo P, Jorgensen RA et al. Betaine, a promising new agent for patients with nonalcoholic steatohepatitis: results of a pilot study. Am J Gastro 2001; 96:2711-2717.
180. Cortez Pinto H, Baptista A, Camilo E et al. Nonalcoholic steatohepatitis clinicopathological comparison with alcoholic hepatitis in ambulatory and hospitalized patients. Dig Dis Sci 1996, 41:172-179.
181. Brunt EM, Janney CG, Di Bisceglie AM et al. Nonalcoholic steatohepatitis: a proposal for grading and staging the histological lesions. Am J Gastroenterol 1999, 94:2467-2474.
182. Brunt EM. Nonalcoholic steatohepatitis: definition and pathology. 2001. Seminars in liver disease 21:3-16.
183. Gunneson TJ, Menon KV, Wiesner RH et al. Ultrasound-assisted percutaneous liver biopsy performed by a physician assistant. Am J Gastro 2002 Jun;97(6):1285-8.
184. Bravo AA, Sheth SG, Chopra S. Liver Biopsy. N Engl J Med 2001;344:495-500
185. Houglum K, Bedossa P, Chojkier M. TGF-beta and collagen-alpha 1 (I) gene expression are

increased in hepatic acinar zone 1 of rats with iron overload. Am J Physiol 1994;267:G908-13.
186. Houglum K, Filip M, Witztum JL et al. Malondialdehyde and 4-hydroxynonenal protein adducts in plasma and liver of rats with iron overload. J Clin Invest 1990;86:1991-8.
187. Palinski W, Yla Herttuala S, Rosenfeld ME et al. Antisera and monoclonal antibodies specific for epitopes generated during oxidative modification of low density lipoprotein. Arteriosclerosis 1990; 10:325-35.
188. Chojkier M, Houglum K, Soli Heruzo J et al. Stimulation of collagen gene expression by ascorbic acid in cultured human fibroblasts. A role for lipid peroxidation? J Biol Chem 1989;264:16 957-62.
189. Ramm GA, Crawford DH, Powell LW et al. Hepatic stellate cell activation in genetic haemo- chromatosis. Lobular distribution, effect of increasing hepatic iron and response to phlebotomy. J Hepatol 1997;26:584-92.
190. Ramm GA, Nair VG, Bridle KR et al. Contribution of hepatic parenchymal and non-parenchymal cells to hepatic fibrogenesis in biliary atresia. Am J Pathol 1998;153:527-35.
191 Friedman S. The cellular basis of hepatic fibrosis. Mechanisms and treatment strategies. N Engl J Med 1993;328:1828-35.
192. MacDonald GA, Bridle KR, Ward PJ et al. Lipid peroxidation in hepatic steatosis in humans is associated with hepatic fibrosis and occurs predominantly in acinar zone 3. J Gastroenterol Hepatol 2001;16:599-606.
193. Olynyk JK, Yeoh GC, Ramm GA et al. Gadolinium chloride suppresses hepatic oval cell proliferation in rats with biliary obstruction. Am J Pathol. 1998;152:347-52.
194. Frisancho AR. New norms of upper limb fat and muscle area for assessment of nutritional status. Am J Clin Nutr 1981; 34:2540-5.
195. Aghdassi E, Tam C, Liu B et al. Body fat of older adult subjects calculated from bioelectric impedance versus anthropometry correlated but did not agree. J Am Diet Ass 2001, 101(10): 27-35.
196. McCrory MA, Fuss PJ, McCallum JE et al. Dietary variety within food groups: association with energy intake and body fatness in men and women. Am J Clin Nutr 1999, 69:440-7.
197. Allard JP, Royall D, Kurian R et al. Effects of beta-carotene supplementation on lipid peroxidation in humans. Am J Clin Nutr 1994, 59:884-90.
198. Allard JP, Aghdassi E, Chau J et al. Effects of vitamin E and C supplementation on oxidative stress and viral load in HIV-infected subjects. AIDS 1998, 12:1653-1659.
199. Hoshino E, Shariff R, Van Gossum A, Allard J et al. Vitamin E suppresses increased lipid peroxidation in cigarette smokers. JPEN 1990;14:300-305.
200. Allard JP, Aghdassi E, Chau J, et al. Oxidative stress and plasma antioxidant micronutrients in humans with HIV infection. Am J Clin Nutr 1998, 67:143-7.
201. Wendland BE, Aghdassi E, Tam C et al. Lipid peroxidation and plasma antioxidant micronutreints in Crohn's disease, Am J Clin Nutr 2001, 74:259-64.
202. Stuff JE, Garza C, O'Brien-Smith E, Nichols BL, Montandon CM. A comparison of dietary methods in nutritional studies. Am J Clin Nutr 1983; 37:300-6.
203. Bessey OA, Lowry OH, Brock MJ. The quantitative determination of ascorbic acid in small amounts of white blood cells and platelets. J Biol Chem 1947;168:197-204.
204. Nata C, Stacewicz-Sapuntzakis M, Bhagavan H, Bowen P. Low serum levels of carotenoids in sickle cell anemia. Eur J Hematol 1988;41:131-135.
205. Sapuntzakis MS, Bowen PE, Kikendall JW, Burgess M. Simultaneous determination of serum retinol and various carotenoids: their distribution in middle-aged men and women. J Micronutr Anal 1987, 3:27-45.
206. Miller NJ, Rice-Evans C, Davies MJ, et al. 1993. Clinical Sciences 84:407-412.
207. Ninfali P, Aluigi G. (1998). Free Radic Res 29:299-408.
208. Dixon JB, Bhathal PS, O'Brien PE. Nonalcoholic fatty liver disease: predictors of nonalcoholic steatohepatitis and liver fibrosis in the severely obese. Gastroenterol 2001;121:91-100.
209. Matthews DR, Hosker JP, Rudenski AS et al. Homeostasis model assessment: insulin resistance and beta-cell function from fasting plasma glucose and insulin concentrations in

man. Diabetologia 1985;28:412-419.
210. Levy JC, Matthews DR, Hermans MP. Correct homeostasis model assessment (HOMA) evaluation uses the computer program [letter]. Diabetes Care;21:2191-92.
211. Katz A, Nambi SS, Mather K et al. Quantitative insulin sensitivity check index: a simple accurate method for assessing insulin sensitivity in humans. J Clin Endocrinol Metab 2000;85:2402-2410.
212. Folch J, Lees M, Sloane-Stanley G. A simple method for the isolation and purification of total lipids from animal tissue. J Biol Chem 1957; 226:497-509.
213. Tocher DR, Bell JG, MacGlaughlin P et al. Hepatocyte fatty acid desaturation and polyunsaturated fatty acid composition of liver in salmonids: effects of dietary vegetal oil. Comp Biochem Physiol 2001, part B 130:257-270.
214. Cherian G, Sim JS. Maternal dietary α-linolenic acid (18:3n-3) alters n-3 polyunsaturated fatty acid metabolism and liver enzyme activity in hatched chicks. Poultry Science 2001;80:901-905.
215. Sadick INS, Hudgins LC. Fatty acid analysis of transplanted adipose tissue. Archives of Dermatology 2001; 137(6):723-727.
216. Allard JP, Kurian R, Aghdassi E. et al. Lipid peroxidation during n-3 fatty acid and vitamin E supplementation in humans. Lipids 1997, 32:535-541.
217. Ma J, Folsom AR, Eckfelt JH et al. Short- and long-term repeatability of fatty acid composition of human plasma phospholipids and cholesterol esters. Am J Clin Nutr 1995, 62:572-78.
218. Nikkari T, Lukkainen P, Pietinen P et al. Fatty acid composition of serum lipid fractions in relation to gender and quality of dietary fat. Annals of Medicine 1995, 27:491-498.
219. Wigg AJ, Roberts-Thomson, Dymock RB, etal. The role of small intestinal bacterial overgrowth, intestinal permeability, endotoxaemia, and tumor necrosis factor alpha in the pathogenesis of non-alcoholic steatohepetitis. Gut 2001; 48:206-211.
220. Sanyal AJ, Cammpbell-Sargent C, Mirshahi F, et al. Nonalcoholic steatohepatitis: association of insulin resistance and mitochondrial abnormalities. Gasteroenterol 2001, 120:1183-1192.
221.. Aghdassi E., Wendland B, Steinhart H, Jeejeebhoy KN, Allard J. Antioxidant vitamin supplementation in Crohn's disease decreases oxidative stress: a randomized controlled trial. Am J Gastro 2002 (in press).

222. Bäckhed F, Ding H et al. The Gut Microbiota as an Environmental Factor That Regulates Fat Storage. PNAS 2004, 101(44): 15718-15723

223. Cani PD, Bibiloni R et al. Changes in Gut Microbiota Control Metabolic Endotoxemia-Induced Inflammation in High-Fat Diet-Induced Obesity and Diabetes in Mice. Diabetes 2008, 57(6): 1470-1481

224. DiBiase JK, Zhang H et al. Gut Microbiota and Its Possible Relationship With Obesity. Mayo Clin Proceed 2008, 83(4): 460-469

225. Dumas ME, Barton RH et al. Metabolic Profiling Reveals a Contribution of Gut Microbiota to Fatty Liver Phenotype in Insulin-Resistant Mice. Proc Natl Acad Sci USA 2006, 103(33):12511-1256.

226. Li Z, Yang S et al. Probiotics and Antibodies to TNF Inhibit Inflammatory Activity and Improve Nonalcoholic Fatty Liver Disease. Hepatology 2003, 37(2): 343-350.

227. Ott SJ, Musfeldt M et al. Quantification of Intestinal Bacterial Populations by Real-Time PCR with a Universal Primer Set and Minor Groove Binder Probes: a Global Approach to the Enteric Flora. J Clin Microbiol 2004, 42(6): 2566-2572
228. Zeisel SH. Gene response elements, genetic polymorphisms and epigenetics influence the human dietary requirement for choline. IUBMB Life 2007;59: 380-387.
229. Zeisel SH, Da Costa KA, Franklin PD, Alexander EA, Lamont JT, Sheard NF, et al. Choline, an essential nutrient for humans. FASEB J 1991;5: 2093-2098.
230. Buchman AL, Ament ME, Sohel M, Dubin M, Jenden DJ, Roch M, et al. Choline deficiency causes reversible hepatic abnormalities in patients receiving parenteral nutrition: proof of a human choline requirement: a placebo-controlled trial. JPEN J Parenter Enteral Nutr 2001;25: 260-268.

231. Buchman AL, Dubin MD, Moukarzel AA, Jenden DJ, Roch M, Rice KM, et al. Choline deficiency: a cause of hepatic steatosis during parenteral nutrition that can be reversed with intravenous choline supplementation. Hepatology 1995;22: 1399-1403.
232. Albright CD, Liu R, Bethea TC, Da Costa KA, Salganik RI, Zeisel SH. Choline deficiency induces apoptosis in SV40-immortalized CWSV-1 rat hepatocytes in culture. FASEB J 1996;10: 510-516.
233. Li Z, Agellon LB, Allen TM, Umeda M, Jewell L, Mason A, et al. The ratio of phosphatidylcholine to phosphatidylethanolamine influences membrane integrity and steatohepatitis. Cell Metab 2006;3: 321-331.
234. Institute of Medicine, National Academy of Sciences. Dietary reference intakes for thiamin, riboflavin, niacin, vitamin B6, folate, vitamin B12, pantothenic acid, biotin and choline. Washington, DC: National Academy Press, 1998.
235. Vance DE: Phospholipid biosynthesis in eukaryotes. In: Vance DE, Vance JE, eds. Biochemistry of Lipids, Lipoproteins and Membranes. Amsterdam: Elsevier Science, 2002; 205-232.
236. Puri P, Baillie RA, Wiest MM, Mirshahi F, Choudhury J, Cheung O, et al. A lipidomic analysis of nonalcoholic fatty liver disease. Hepatology 2007;46: 1081-1090.
237. Li Z, Vance DE. Phosphatidylcholine and choline homeostasis. J Lipid Res 2008;49: 1187-1194.
238. Yao ZM, Vance DE. The active synthesis of phosphatidylcholine is required for very low density lipoprotein secretion from rat hepatocytes. J Biol Chem 1988;263: 2998-3004.
239. Devaux PF. Static and dynamic lipid asymmetry in cell membranes. Biochemistry (Mosc). 1991;30: 1163-1173.
240. Claus SP, Tsang TM, Wang Y, Cloarec O, Skordi E, Martin FP, et al. Systemic multicompartmental effects of the gut microbiome on mouse metabolic phenotypes. Mol Syst Biol 2008;4: 219.
241. Bligh EG, Dyer WJ. A rapid method of total lipid extraction and purification. Canadian Journal of Biochemistry and Physiology 1959;37: 911-917.
242. Koc H, Mar MH, Ranasinghe A, Swenberg JA, Zeisel SH. Quantitation of choline and its metabolites in tissues and foods by liquid chromatography/electrospray ionization-isotope dilution mass spectrometry. Anal. Chem. 2002;74: 4734-4740.
243. Khachatryan ZA, Ktsoyan ZA, Manukyan GP, Kelly D, Ghazaryan KA, Aminov RI. Predominant role of host genetics in controlling the composition of gut microbiota. PLoS One 2008;3:e3064.
244. Mai V. Dietary modification of the intestinal microbiota. Nutr. Rev. 2004;62:235-242.
245. Eckburg PB, Bik EM, Bernstein CN, Purdom E, Dethlefsen L, Sargent M, et al. Diversity of the Human Intestinal Microbial Flora. Science 2005;308:1635-1638.
246. Hayashi H, Sakamoto M, Benno Y. Phylogenetic Analysis of the Human Gut Microbiota Using 16S rDNA Clone Libraries and Strictly Anaerobic Culture-Based Methods. Microbiol. Immunol. 2002;46:535-548.
247. Hold GL, Pryde SE, Russell VJ, Furrie E, Flint HJ. Assessment of microbial diversity in human colonic samples by 16S rDNA sequence analysis. FEMS Microbiol. Ecol. 2002;39:33-39.
248. Zhang H, DiBaise JK, Zuccolo A, Kudrna D, Braidotti M, Yu Y, et al. Human gut microbiota in obesity and after gastric bypass. Proc Natl Acad Sci U S A 2009;106:2365-2370.
249. Hill MJ. Composition and control of ileal contents. Eur. J. Cancer Prev. 1998;7:S75-78.
250. Zhang C, Zhang M, Wang S, Han R, Cao Y, Hua W, et al. Interactions between gut microbiota, host genetics and diet relevant to development of metabolic syndromes in mice. ISME J;4:232-241.
251. Moore WE, Moore LH. Intestinal floras of populations that have a high risk of colon cancer. Appl. Environ. Microbiol. 1995;61:3202-3207.
252. Mai V, Colbert LH, Perkins SN, Schatzkin A, Hursting SD. Intestinal microbiota: A potential diet-responsive prevention target in ApcMin mice. Mol. Carcinog. 2007;46:42-48.
253. DiBaise JK, Zhang H, Crowell MD, Krajmalnik-Brown R, Decker GA, Rittmann BE. Gut microbiota and its possible relationship with obesity. Mayo Clin Proc 2008;83:460-469.
254. Samuel BS, Gordon JI. A humanized gnotobiotic mouse model of host-archaeal-bacterial mutualism. Proc Natl Acad Sci U S A 2006;103:10011-10016. Epub 12006 Jun 10016.
255. Turnbaugh PJ, Ley RE, Mahowald MA, Magrini V, Mardis ER, Gordon JI. An obesity-associated gut microbiome with increased capacity for energy harvest. Nature 2006;444:1027-1031.
256. Ley RE, Backhed F, Turnbaugh P, Lozupone CA, Knight RD, Gordon JI. Obesity alters gut microbial ecology. Proc Natl Acad Sci U S A 2005;102:11070-11075.
257. Siebler J, Galle PR, Weber MM. The gut-liver-axis: Endotoxemia, inflammation, insulin resistance and NASH. J Hepatol 2008;48:1032-1034.

258. Cani PD, Amar J, Iglesias MA, Poggi M, Knauf C, Bastelica D, et al. Metabolic endotoxemia initiates obesity and insulin resistance. Diabetes 2007;56:1761-1772.
259. Song MJ, Kim KH, Yoon JM, Kim JB. Activation of Toll-like receptor 4 is associated with insulin resistance in adipocytes. Biochem Biophys Res Commun 2006;346:739-745.
260. Suganami T, Mieda T, Itoh M, Shimoda Y, Kamei Y, Ogawa Y. Attenuation of obesity-induced adipose tissue inflammation in C3H/HeJ mice carrying a Toll-like receptor 4 mutation. Biochem Biophys Res Commun 2007;354:45-49.
261. Tsukumo DM, Carvalho-Filho MA, Carvalheira JB, Prada PO, Hirabara SM, Schenka AA, et al. Loss-of-function mutation in Toll-like receptor 4 prevents diet-induced obesity and insulin resistance. Diabetes 2007;56:1986-1998.
262. Cani PD, Delzenne NM. The role of the gut microbiota in energy metabolism and metabolic disease. Curr. Pharm. Des. 2009;15:1546-1558.
263. Shi H, Kokoeva MV, Inouye K, Tzameli I, Yin H, Flier JS. TLR4 links innate immunity and fatty acid-induced insulin resistance. J Clin Invest 2006;116:3015-3025.
264. Hamer HM, Jonkers D, Venema K, Vanhoutvin S, Troost FJ, Brummer RJ. Review article: the role of butyrate on colonic function. Aliment Pharmacol Ther 2008;27:104-119.
265. Cani PD, Bibiloni R, Knauf C, Waget A, Neyrinck AM, Delzenne NM, et al. Changes in gut microbiota control metabolic endotoxemia-induced inflammation in high-fat diet-induced obesity and diabetes in mice. Diabetes 2008;57:1470-1481.
266. Poggi M, Bastelica D, Gual P, Iglesias MA, Gremeaux T, Knauf C, et al. C3H/HeJ mice carrying a toll-like receptor 4 mutation are protected against the development of insulin resistance in white adipose tissue in response to a high-fat diet. Diabetologia 2007;50:1267-1276.
267. Zeisel SH. Gene response elements, genetic polymorphisms and epigenetics influence the human dietary requirement for choline. IUBMB Life 2007;59:380-387.
268. Buchman AL, Ament ME, Sohel M, Dubin M, Jenden DJ, Roch M, et al. Choline deficiency causes reversible hepatic abnormalities in patients receiving parenteral nutrition: proof of a human choline requirement: a placebo-controlled trial. JPEN J Parenter Enteral Nutr 2001;25:260-268.
269. Buchman AL, Dubin MD, Moukarzel AA, Jenden DJ, Roch M, Rice KM, et al. Choline deficiency: a cause of hepatic steatosis during parenteral nutrition that can be reversed with intravenous choline supplementation. Hepatology 1995;22:1399-1403.
270. Li Z, Agellon LB, Allen TM, Umeda M, Jewell L, Mason A, et al. The ratio of phosphatidylcholine to phosphatidylethanolamine influences membrane integrity and steatohepatitis. Cell Metab 2006;3:321-331.
271. Dumas ME, Barton RH, Toye A, Cloarec O, Blancher C, Rothwell A, et al. Metabolic profiling reveals a contribution of gut microbiota to fatty liver phenotype in insulin-resistant mice. Proc Natl Acad Sci U S A 2006;103:12511-12516.
272. Claus SP, Tsang TM, Wang Y, Cloarec O, Skordi E, Martin FP, et al. Systemic multicompartmental effects of the gut microbiome on mouse metabolic phenotypes. Mol Syst Biol 2008;4:219.
273. Thuy S, Ladurner R, Volynets V, Wagner S, Strahl S, Konigsrainer A, et al. Nonalcoholic fatty liver disease in humans is associated with increased plasma endotoxin and plasminogen activator inhibitor 1 concentrations and with fructose intake. J Nutr 2008;138:1452-1455.
274. Jiang W, Lederman MM, Hunt P, Sieg SF, Haley K, Rodriguez B, et al. Plasma levels of bacterial DNA correlate with immune activation and the magnitude of immune restoration in persons with antiretroviral-treated HIV infection. J. Infect. Dis. 2009;199:1177-1185.
275. Hastie T, Tibshirani R, Friedman J. The Elements of Statistical Learning: Data Mining, Inference and Prediction. New York: Springer, 2003.
276. Turnbaugh PJ, Hamady M, Yatsunenko T, Cantarel BL, Duncan A, Ley RE, et al. A core gut microbiome in obese and lean twins. Nature 2009;457:480-484.
277. Rodriguez-Brito B, Rohwer F, Edwards R. An application of statistics to comparative metagenomics. BMC Bioinformatics 2006;7:162.
