# Supplementary material for: Altered hepatic genes related to retinol metabolism and plasma retinol in patients with non-alcoholic fatty liver disease
Source: PLoS One. 2018 Oct 31;13(10):e0205747. doi: 10.1371/journal.pone.0205747 (PMC6209208; doi:10.1371/journal.pone.0205747)
Supplement: S3 File — (PDF) [file pone.0205747.s003.pdf]

Supplementary Methods

Hepatic gene expression analysis

RNA extraction: Total RNA was extracted from liver biopsies using the *mir*VanaTM miRNA Isolation kit (Life Technologies Corp., Carlsbad, CA, USA) as per the manufacturer's instructions, eluted in 100 µl of RNAse-free water and stored at -80°C. RNA concentration and purity were assessed with a Thermo Scientific's NanoDrop 1000 Spectrophotometer (NanoDrop Technologies, Wilmington, DE, USA) and quality was assessed with an Agilent 2100 Bioanalyzer (Agilent, Palo Alto, CA, USA).

Analysis on Illumina Platform: Two hundred nanograms RNA were labelled in a randomized fashion and amplified following Illumina Whole Genome Gene Expression DASL Assay kit for partially degraded and FFPE derived RNA (Illumina Inc., San Diego, CA, USA). The labelled samples were then hybridized onto six Human HT-12 V4 BeadChips. The BeadChips were incubated at 58°C, for 18 h with rotation speed 5 for hybridization. The BeadChips were then washed and coated as per Illumina protocol and scanned on the iScan (Illumina). The data files were quantified in GenomeStudio Version 2011.1 (Illumina).

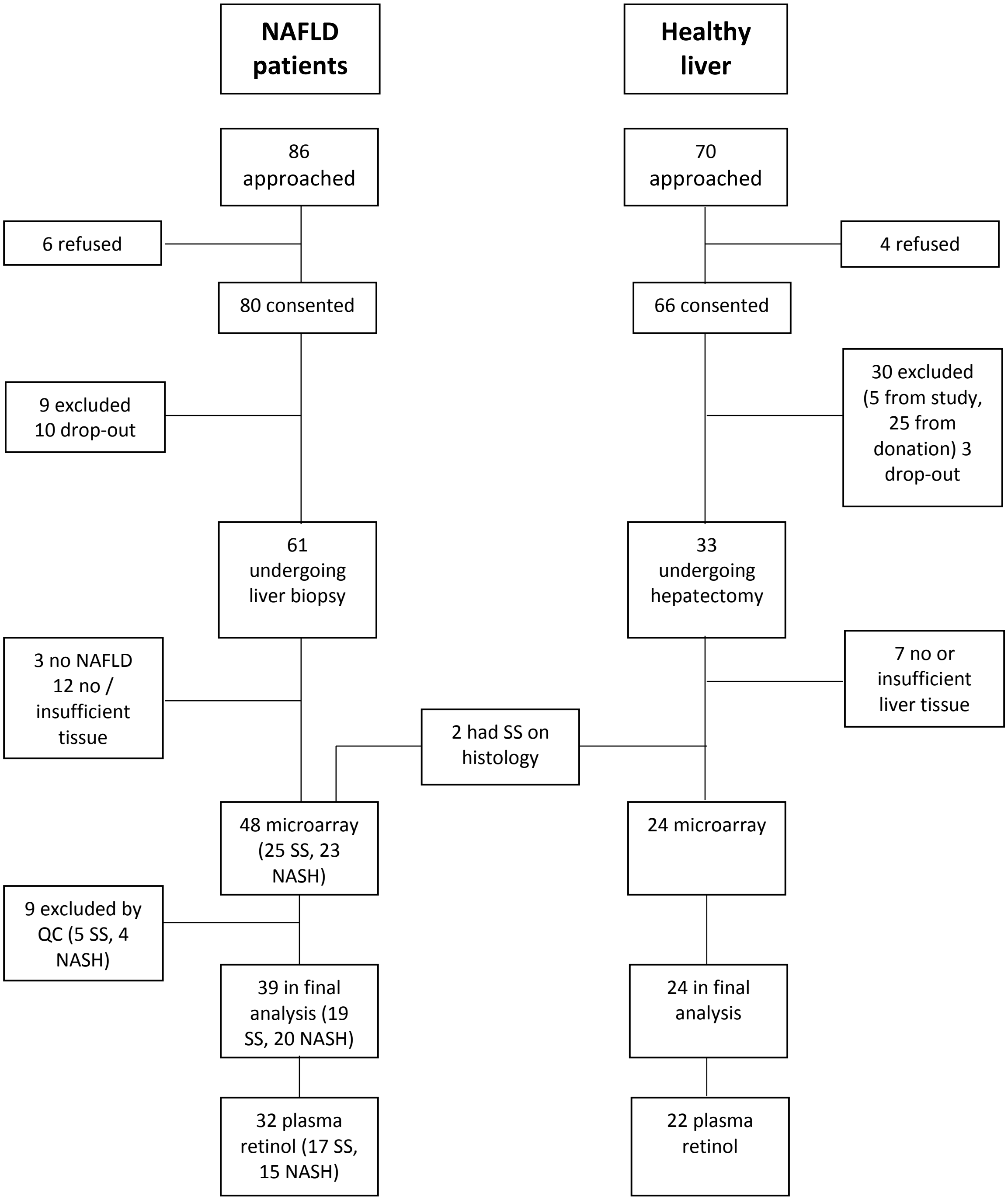

Supplementary Figure. Participant Flow
